# Developing a Theoretically Informed Implementation Model for Telemedicine-Delivered Medication for Opioid Use Disorder: Qualitative Study With Key Informants

**DOI:** 10.2196/47186

**Published:** 2023-10-18

**Authors:** Joseph Tay Wee Teck, Rosalind Gittins, Giedre Zlatkute, Alberto Oteo Pérez, Susanna Galea-Singer, Alexander Baldacchino

**Affiliations:** 1 Digital Health Interventions in Addiction Services Project, Population and Behavioural Science School of Medicine University of St Andrews St Andrews United Kingdom; 2 Via London United Kingdom; 3 NHS Fife Addiction Services Leven United Kingdom

**Keywords:** telemedicine, medication for opioid use disorder, implementation model, telebuprenorphine, opioid use disorder, mobile phone

## Abstract

**Background:**

Telemedicine-delivered medication for opioid use disorder (TMOUD) has become more prevalent during the COVID-19 pandemic, particularly in North America. This is considered a positive development as TMOUD has the potential to increase access to evidence-based treatment for a population heavily affected by the opioid crisis and consequent rising mortality and morbidity rates in relation to opioid use disorder. Despite the increase in the use of TMOUD, there are no established service- and process-focused models to guide the implementation of this intervention.

**Objective:**

This study aims to develop a process- and service-focused implementation model in collaboration with key stakeholders and bring together peer-reviewed literature, practice-based knowledge, and expert opinions.

**Methods:**

The simple rules for evidence translation in complex systems framework was applied to guide the development of a 6-step qualitative study. The steps were definition of the scope and objectives of the model, identification of evidence, stakeholder engagement, draft model development, key informant consultation, and final model specification.

**Results:**

The final specification for the TMOUD implementation model incorporated key strategic priorities, service delivery prerequisites, service design elements, stakeholder identification and engagement, key process domains, and iterative cycles of evaluation and improvement.

**Conclusions:**

Through stakeholder engagement and key informant consultation, we produced a process- and service-focused TMOUD implementation model. The model is modifiable to different contexts and settings while also in keeping with the current evidence base and national and international standards of high-quality opioid use disorder care.

## Introduction

### Background

Scotland has been experiencing a rising trend in drug-related deaths (DRDs) for 2 decades [1]. In 2020, the per capita DRD rate in Scotland was 245 deaths per million population, the highest in the European Union and approaching US rates of 277 deaths per million population [2]. In June 2019, the Scottish government established the Drug Deaths Task Force to identify policies, measures, and interventions that could reduce DRDs and harms [3]. A key task force recommendation was the introduction of evidence-based standards to enable the delivery of high-quality, safe, timely, and accessible drug treatments across Scotland, incorporating medication for opioid use disorder (MOUD) [4].

The Scottish government’s decision to improve the provision and quality of MOUD was in line with the international evidence base and priorities for reducing DRDs [5]. MOUD, such as methadone and buprenorphine, can notably reduce overdose risk and associated deaths, thus improving health and social outcomes, particularly when patients remain on treatment [6,7]. Nevertheless, internationally, only 1 in every 6 people seeking treatment for opioid use disorder (OUD) receives it [8]. Several factors contribute to this so-called treatment gap [9], including stigma [10], limited clinical resources, insufficient investment, ambivalence toward and difficulties accessing MOUD [11-14], and stringent regulatory barriers [15]. Furthermore, vulnerable groups such as younger people [16], pregnant women, mothers [17,18], rural residents [19], racial and ethnic minority groups [20], and people experiencing displacement [21,22] or homelessness [23] disproportionately struggle to access MOUD. Consequently, the Scottish government’s first 5 standards for medication-assisted treatment (MAT), which focuses on MOUD, include provision for same-day access, choice of medication, assertive outreach and anticipatory care, harm reduction, and retention in treatment [24] (Textbox 1).

**Scottish medication-assisted treatment (MAT) standards 1 to 5, which focus on the provision of medication for opioid use disorder.**

**Standard 1**

- All the people accessing services have the option to start MAT from the same day of presentation.

**Standard 2**

- All the people are supported to make an informed choice on what medication to use for MAT and the appropriate dose.

**Standard 3**

- All the people at high risk of drug-related harm are proactively identified and offered support to commence or continue MAT.

**Standard 4**

- This standard comprises the number of services offering evidence-based harm reduction at the point of MAT delivery.

**Standard 5**

- All the people will receive support to remain in treatment for as long as requested.

To embed these standards across Scotland by April 2022, the Scottish government funded several demonstration projects, a research program, and an innovation fund to address gaps in knowledge and service delivery [25]. The COVID-19 pandemic created significant disruptions to this work program [25] yet created the opportunity for an exploration of the use of telemedicine in delivering MOUD. Telemedicine-delivered MOUD (TMOUD) has shown promise in addressing the treatment gap by increasing immediacy and responsiveness; reducing geographical and logistical barriers, stigma, and costs; enabling more consistent and regular patient contact; and increasing service efficiency [26-31]. In response to service closures, reduced staffing capacity, and the introduction of measures to reduce COVID-19 transmission, some countries introduced regulatory, funding, and clinical governance changes allowing for TMOUD to be used more widely [32-37]. This was particularly the case in the United States and Canada, where a rapid acceleration of TMOUD adoption was observed [32].

In October 2020, the Digital Health Interventions in Addiction Services project at St Andrews University received funding through the Scottish Drug Deaths Taskforce and the Corra Foundation to explore ways of developing and implementing TMOUD as part of high-quality drug treatment in Scotland. This project was timely as we were able to observe and learn from international experiences with TMOUD implementation in response to the COVID-19 pandemic. Indeed, an early finding from the accelerated and necessary transitions to telemedicine to provide MOUD was that it created uncertainty and anxiety among clinicians and service providers in a variety of settings and countries [36,38]. This largely regarded issues related to governance; regulatory compliance; safety; confidentiality; and best practice approaches to history taking, physical examination, and investigations such as drug and blood-borne virus testing [39].

### Objectives

From an implementation science perspective, such contextual, operational, and clinical concerns are key pivot points determining the success or failure of new TMOUD services [40]. However, to our knowledge, there are no specific implementation frameworks or models to facilitate the embedding of TMOUD into standard drug treatment practice. The literature on TMOUD before the COVID-19 pandemic tended to comprise retrospective data analyses comparing the efficacy of in-person versus telemedicine delivery of MOUD [41], with a steep rise in publications from 2020 onward describing pandemic-related rapid service transitions to TMOUD [32]. Consequently, our aim with this work was to describe the development of a theoretically informed TMOUD implementation model.

## Methods

### Setting

Addiction treatment services are complex systems of care with multiple interdependent components and autonomous actors whose agency in performing their everyday roles can hamper or disrupt attempts to introduce sustainable change [42-44]. Furthermore, the delivery of MOUD both in Scotland and internationally occurs within a highly regulated clinical guidance, governance, and legislative framework associated with a high level of scrutiny on compliance from both people with OUD seeking treatment and treatment providers [45]. In the context of this TMOUD project, at least 3 additional layers of intersecting complexity were identified.

First, initial actions in 2020 to introduce and implement quality standards for MOUD delivery in Scotland were enabling in nature, involving stakeholder engagement, quality improvement support, funding for innovative pilot projects, development of processes and tools, and their wide dissemination [24]. Unfortunately, a progress report in March 2021 [24], echoed by a national benchmarking process [46], identified significant barriers to services achieving these standards, including challenges with leadership and financial planning, conflicting local priorities, and a lack of transparency and evaluation data [24,46]. This culminated in the Scottish government using legislative powers to compel health boards, integration authorities, and local authorities to drive the changes necessary to deliver on these quality standards, particularly same-day access to MOUD [47]. Improving access to and retention of MOUD will inevitably result in growing numbers in treatment, and services will need to adapt, evolve, and innovate to provide ongoing, safe, and effective care. Both the United States and Canada, with similar drug death crises, have developed models of TMOUD to improve service efficiency and the capacity to expand and meet the demand for treatment [48].

Second, Scotland has the necessary telemedicine-specific infrastructure, policy, and governance frameworks for TMOUD implementation. Specifically, there has been a strategic development plan for telemedicine and telecare in Scotland since 2012 [49] and an established platform to deliver telemedicine through the Attend Anywhere/Near Me video consulting service since 2016 supported by an implementation framework provided by Technology Enabled Care Scotland [50]. Indeed, this background work meant that Scotland was able to successfully introduce a rapid rollout initiative of video consulting in the context of the COVID-19 pandemic [51]. Unfortunately, addiction services consistently lagged behind other health specialties in using the Near Me video consulting service both before and after the rapid rollout initiative. When compared with psychiatry, psychology, and community mental health services, for example, addiction services made up 0.77% (44/5745) versus 9.5% (546/5745) of telemedicine consultations from January 2019 to December 2019 [50], and this number reduced further after the rapid rollout in response to the pandemic with 0.25% (651/260,547) in contrast to 36.41% (94,876/260,547) [52] of telemedicine consultations. Nevertheless, although the Scottish government mandated quality standards for MOUD delivery, there were no targeted financial or policy drivers to incentivize the uptake of TMOUD.

Third, the peer-reviewed evidence base for TMOUD has expanded greatly and continued to grow since the COVID-19 pandemic [48]. There were nearly 4 times more peer-reviewed publications on TMOUD in January 2023 compared with before 2020 [48]. This evidence base is dominated by US-based research, which differs in critical ways from the Scottish and UK contexts [48]. Key differences include a highly restricted approach to methadone provision and the preponderance of buprenorphine as a first-line MOUD in the United States as opposed to the United Kingdom, where both medications are available at community pharmacies with a roughly 60:40 split in favor of methadone [53]. Consequently, it was necessary to find ways to adapt existing knowledge to local processes and iteratively review the growing evidence base as knowledge gaps emerged throughout this project.

### Theoretical Underpinning: Applying the Successful Healthcare Improvements From Translation of Evidence Into Practice Framework

The setting of this TMOUD project necessitated a theoretical framework suited to managing complexity, and the Successful Healthcare Improvements From Translation of Evidence Into Practice (SHIFT-Evidence) framework was identified as particularly comprehensive in this regard [54]. This framework describes 3 strategic principles and 12 simple rules on how to make sense of and intervene in complex systems with a focus on service delivery and processes [54]. The first strategic principle, to act scientifically and pragmatically, is an exhortation to tailor and iteratively adapt implementation approaches to match local contexts, problems, and opportunities [54]. The second principle, to embrace complexity, essentializes the need to fully understand usual care practices and processes and identify and address existing and emergent issues through evidence translation efforts [54]. The third principle, to engage and empower, highlights the importance of aligning evidence translation with the concerns and motivations of the people who underpin, perform, and engage with real-world processes and practices [54]. Associated with these 3 principles are 12 simple rules that have been applied to design this study, described in detail in Multimedia Appendix 1.

### Research Design

We used a logic model [55] to describe the overall approach taken to develop this TMOUD implementation model (Multimedia Appendix 2). The logic model is based on the application of the 12 SHIFT-Evidence framework rules detailed in Multimedia Appendix 1.

#### Step 1: Define the Scope and Objectives of the Implementation Model

This model was intended to focus on process development and improvement (eg, workflows, role allocation, infrastructure, and resources) to facilitate TMOUD implementation rather than define ideal clinical standards or predict or analyze barriers and facilitators. A key tenet of the project was to promote a participatory approach such that the implementation model produced would be informed by clinical expertise, professional values, and obligations; customizable to specific contexts; and implementable through collective effort rather than a top-down imposition. In keeping with theories that explain the “kinds of work” necessary to successfully implement an intervention, such as normalization process theory [56], the model needed to characterize TMOUD as distinct from standard MOUD practices, identify critical stakeholder relationships and perspectives, and describe necessary processes and actions and appraisal work for iterative improvements [56].

#### Step 2: Identification of Evidence to Inform the Implementation Model

As discussed previously, the evidence base for TMOUD has been rapidly evolving and growing, and implementation- or practice-specific knowledge has been lacking. We addressed this issue by carrying out a series of scoping reviews [48,57] that are flexible, inclusive, and iterative in nature [58] along with the sourcing of expert case studies through addiction professional networks such as the International Society of Addiction Medicine and the Canadian Society of Addiction Medicine.

#### Step 3: Stakeholder Identification and Engagement

In Scotland, local-level drug and alcohol strategy and policy implementation is devolved to 31 Alcohol and Drug Partnerships (ADPs), which work across statutory and third-sector health and social care providers. We identified stakeholders through ADPs but also through national advocacy organizations; drug and alcohol research networks; and professional organizations such as the Royal College of Psychiatrists, Royal College of General Practitioners, College of Mental Health Pharmacy, and the Royal Pharmaceutical Society. As the use of TMOUD was not prominent on the national agenda and local initiatives were nonexistent, we launched the participatory component of this research through a web-based engagement event linking stakeholders with an international panel of TMOUD experts. Each presenter was a service provider from the United States, Canada, England, Ireland, or Scotland and provided a 10-minute presentation on their experiences in implementing aspects of TMOUD. A summary of the topics and content covered is provided in Multimedia Appendix 3, and recordings of the presentations are available [59]. Stakeholders were able to engage in discussions with expert panelists in a synchronous chat. Participants at the engagement event were invited to indicate whether they wished to take part in the future TMOUD implementation model development.

#### Step 4: Produce a Draft Implementation Model for Consultation

The first iteration of the draft implementation model was based on a review of policy documents [4,60,61], information collected from our expert panel, synchronous discussion transcripts from the engagement seminar, and scoping reviews conducted to identify examples of program implementation and models and processes underpinning TMOUD delivery [48,57].

#### Step 5: Key Informant (Stakeholder) Consultation

The draft implementation model was made available in an electronic PDF file (Multimedia Appendix 4) as well as an interactive web-based collaborative document (Multimedia Appendix 5). Participants from the stakeholder engagement event who had indicated an interest in commenting on the draft model were invited to sign up to do this via a unique link to the web-based collaboration platform. This web-based platform allowed participants to provide asynchronous comments on the same interactive document, adding a layer of transparency to the overall process. This was not convenient for all our participants as some could not access the web-based platform owing to organizational firewalls, whereas others preferred to retain a degree of anonymity when commenting. A PDF copy of the draft document was emailed to these participants if so requested. Comments, conflicts, and proposed amendments were incorporated into a revised document, which was then discussed at a final roundtable meeting with key implementation decision makers and leaders.

#### Step 6: Produce a Final Specification of the Implementation Model

Following the key informant and stakeholder consultation process, the updated document was combined with additional findings of a literature review carried out throughout the research period. This confluence of research and practice-based evidence and expert opinions formed the final specification of the TMOUD implementation model.

### Ethics Approval

Ethics approval was granted by the St Andrews University Teaching and Research Ethics Committee (MD15635).

## Results

### Key Informant (Stakeholder) Consultation

There was diverse representation across key stakeholder groups in our initial engagement event and subsequent participatory activities. A total of 264 participants logged on to the engagement event, including National Health Service representatives (n=50, 18.9%), third-sector care providers (n=42, 15.9%), participants from policy and patient advocacy organizations and international nongovernmental organizations, pharmaceutical representatives, non-UK addiction care providers, participants from the Scottish government, and ADP representatives. A total of 10.6% (28/264) of the people attending the engagement event actively interacted with the panelists through chat room discussions. In total, 71 participants were invited to review the first draft of the implementation model, with 18 (25%) representing diverse stakeholders actively contributing. A total of 5.7% (15/264) of the participants took part in the final roundtable discussion. This roundtable meeting was focused on obtaining input from decision makers and clinical leaders on gaps and vulnerabilities within the draft implementation model. Multimedia Appendix 6 details the stakeholder roles represented in each of the participatory activities we conducted.

### Final Specification of the TMOUD Implementation Model

**Figure 1:**
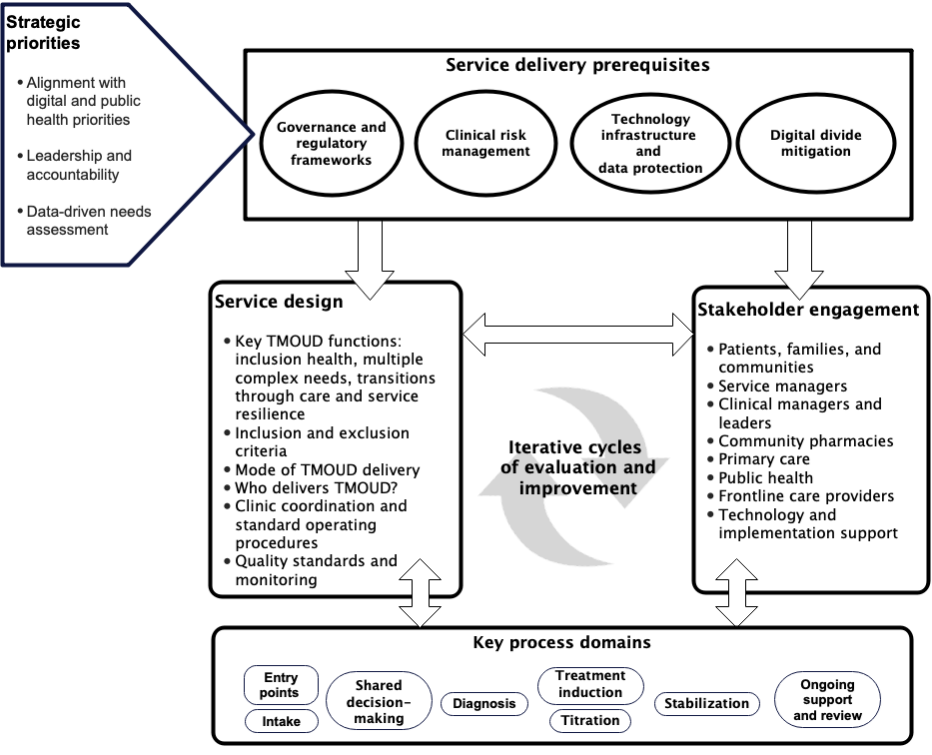
Telemedicine-delivered medication for opioid use disorder (TMOUD) implementation model.

Figure 1 provides a graphical representation of the TMOUD implementation model. The key components of the model were strategic priorities, service delivery prerequisites, service design factors, stakeholder engagement, key process domains, and iterative cycles of evaluation and improvement.

#### Strategic Priorities

##### Strategic Alignment With Regional Digital Health Policy and Public Health Priorities

There was broad recognition among key informants that prospective TMOUD projects brought together the public health priority of reducing DRD rates and national digital health strategies. Critically, local innovation ecosystems are frequently linked to national digital health policy, and an understanding of these by addiction service providers can facilitate the internal buy-in of TMOUD implementation processes. More specifically, significant investments in digital infrastructure, training, and other resources were readily available as a consequence of national digital health policies [61], ready to support local TMOUD adoption. Some key informants believed that supporting the engagement of addiction services and clinicians with other specialties such as urgent care, which was already making use of telemedicine, and introducing telemedicine champions and technology training programs may help build confidence in TMOUD implementation.

##### Leadership and Accountability

Leadership and strategic direction were identified as critically important to the implementation of TMOUD [48,62-64]. For example, early guidance from the Irish College of General Practitioners in conjunction with the National Health Service Executive Office for Social Inclusion [65] reassured clinicians who were concerned about divergence from standard in-person practice when delivering TMOUD [65]. One of the key informants with telemedicine implementation experience reinforced the value of organizational-level clinical leadership to gain an understanding of strengths, infrastructure, and risks; adapt a generic telemedicine model to the needs of the local service providers and population; and engage frontline clinicians in TMOUD processes, management, and delegation, a perspective supported by the literature [62,64,66].

##### Data-Driven Needs Assessment

In total, 2 areas requiring a needs assessment were highlighted. The first was to understand the geographic distribution of people who use drugs, including travel time to service, availability of a dispensing pharmacy, transportation availability and costs, broadband coverage, service and clinician capacity, and the person’s preference for contact type. Some of these data may be readily available from existing geo-mapping initiatives, whereas other data may require active engagement with local communities and auditing the service. The second aspect involved understanding the needs of local people and what may be missed when a service is transferred to telemedicine. Several examples of this were provided by key informants, for example, blood-borne virus testing and treatment, sexual health, housing support, overdose awareness, and take-home naloxone provision. Specifically, TMOUD services need to consider how more holistic aspects of drug treatment services can continue within a telemedicine model of care.

#### Service Delivery Prerequisites

##### Governance and Regulatory Frameworks

Variability in jurisdictional legal and professional regulatory frameworks and local guidelines and practice regarding the prescription of controlled drugs such as methadone and buprenorphine may act as barriers to TMOUD implementation [63,66]. These frameworks are not only often legally mandated but are also often perceived as synonymous with good clinical practice [67]. Examples of regulatory restrictions that require particular attention in the development of TMOUD include regional prescription drug monitoring programs to limit the risk of inappropriate provision of a medication that could cause dependency and mandatory in-person consultations before the provision of MOUD [27,68,69]. However, growing evidence has shown that overregulation results in barriers to MOUD provision, pushing some patients into the illicit market to manage opioid withdrawal [70].

To mitigate the impact of the COVID-19 pandemic, many jurisdictions introduced changes to regulatory, funding, and clinical governance policies regarding MOUD delivery [71]. Areas that allowed more extensive regulatory easing and policy flexibility also had more developed and successful TMOUD implementation [30,32,36,37,64]. For example, because of nonnegotiable prescribing arrangements for several controlled drugs, including methadone, India was unable to implement TMOUD in any meaningful way [38] compared with its North American counterparts [27,64,69,72]. Other important regulatory considerations include reimbursement arrangements and payment parity between telemedicine and in-person consultations [27,68,69,73,74], information governance safeguards [38,68], the availability of sanctioned telemedicine platforms [66,75], and medical indemnity coverage for physicians [62,68].

It follows, then, that an overarching priority must be for services to identify the policy and legislative limits to TMOUD implementation and whether temporary or enduring exemptions or memoranda of understanding are in place or negotiable. For example, the US TMOUD expansion throughout the COVID-19 pandemic was almost certainly facilitated by payment parity approvals for telemedicine by health insurance companies [32], permission for a broad range of videoconferencing tools to be used for telemedicine, and waiving the requirement for an in-person examination before controlled substances were prescribed [75]. Addiction care providers in the United States have been keen to document the value of pandemic-related regulatory easing in narrowing inequalities to MOUD access and the OUD treatment gap [66] and work to sustain the environment that made TMOUD implementation so successful [76]. A similar advocacy approach may be valuable to other jurisdictions keen to develop locally appropriate TMOUD services.

One key informant highlighted that addiction care providers may not always fit within the same governance framework; thus, the limitations, barriers, and facilitators may not be congruent. For example, in Scotland, taxpayer-funded and statutorily governed National Health Service addiction care providers use a single validated secure communications platform and have specific reimbursement arrangements for MOUD [77]. In England, it is often third-sector organizations that are commissioned by local authorities to provide addiction services, each having its own negotiated service-level contracts and funding arrangements [78]. Similarly, in Canada, the decentralization of methadone regulation to provinces in 1995 allowed each jurisdiction to adapt according to local needs and geographic and staffing practices, resulting in some areas, such as Ontario, being able to deliver TMOUD since 2013 [26]. However, in the United States, the complexities of local variations in legislation and reimbursement policies, together with overarching federal laws such as the Ryan Haight Online Pharmacy Consumer Protection Act of 2008, have resulted in provider organizations having to expend considerable effort to ensure they do not inadvertently break the law when providing TMOUD [68].

##### Clinical Risk Management

Clinical risk management (CRM) is the process by which health care organizations ensure the quality and safety of the services they provide, identify circumstances that contribute to clinical risk, and act to prevent or control those risks [79]. CRM is typically described as a 4-step process starting with risk identification, analysis of severity and frequency, development and application of a reduction or elimination plan, and iterative evaluation of the impact of this plan on risk reoccurrence [79]. Although CRM is a critical component of TMOUD service design and delivery, 2 main concerns were highlighted. The first was the way in which bureaucratic risk responses by prescribers derived from organizational CRM may widen the digital divide and structural inequalities [48]. Specifically, a scoping review of TMOUD highlighted the practice of only allowing telemedicine to be used for the delivery of MOUD to people who were already established in treatment, excluding those who were new to the service as the perception was that the latter group posed a high risk of indemnity if not seen in person at the outset [48]. This was despite the regulatory easing that sanctioned buprenorphine induction without an initial in-person visit [80]. The outcome of this in the United States was the exclusion of marginalized and ethnic minority groups from accessing MOUD as they were not already within the treatment system at the pandemic onset. Critically, these groups did not have a mechanism to challenge this decision or highlight the increased risks they experienced as a result [48].

The second issue was related to prescriber attitudes toward risk, which are typically influenced by social relationships, attitudes, trust, values, power dynamics and hierarchies, experience, and knowledge [48]. Consequently, providing evidence of good outcomes or mandating the delivery of TMOUD without addressing the local risk culture would be unlikely to enable successful implementation. A theme among the key informants was the importance of ongoing engagement with stakeholders, including people with OUD, in the design of TMOUD services, risk assessments, and CRM activities. Specifically, stakeholders should be involved in the iterative evaluation of the impact of CRM in reducing risks to individuals in the context of digitally delivered care rather than assuming that the situation remains the same as with in-person care. Consequently, the cornerstone of CRM is a full understanding of existing MOUD processes and the associated or potential risks when transitioning to TMOUD, an area described in detail in the *Key Process Domains* section.

##### Technology Infrastructure and Data Protection

Issues such as dropped calls, poor-quality images or sound, time lag disrupting the flow of communication, and fluctuating access to bandwidth that interferes with clinical consultations are common in telemedicine consults [36,38,80,81]. Service providers have needed to transfer the consultation to a telephone call in some cases, potentially limiting the amount of information available to support diagnosis and treatment [36,38,80,81]. Furthermore, although the Scottish National Health Service has had access to a nationally approved telemedicine platform with appropriate governance and security measures in place [60], this is not the case for many nonstatutory service providers, who have relied on commercial services such as Skype for business or encrypted Zoom calls. Each of these platforms may have different privacy law compliance and may differ in their integration with electronic health records. Several key informants recommended dedicated resources to ensure that the technology underpinning telemedicine was robustly tested and that troubleshooting support was readily available. Clear guidance and reassurance should be provided to clinicians that consultation platforms are compliant with data protection legislation.

##### Digital Divide Mitigation

There was an awareness of and efforts to mitigate the digital divide in Scotland [82-84], which encompassed addressing technical skills, confidence, broadband and data poverty, and equipment access and affordability. Some patients with OUD experiencing homelessness may struggle to safeguard or charge devices, find the privacy to join a telemedicine consult, or have the “headspace” to engage with technology because of other more immediate life-critical priorities [85]. Several initiatives to address these more complex barriers to TMOUD were identified, including fixed or mobile telemedicine pods, using bridge or link workers as telemedicine support, and diversifying digital options to include telephone contact.

Mitigation strategies should include an awareness of how clinical practice or policies may directly widen the digital divide. For example, concerns have been raised over audio-only versus video-based telemedicine being less efficient and effective [86]. Regulators in the United States are considering mandating against audio-based TMOUD despite better engagement of people at the extreme margins of society [87,88]. However, the implication for developing video-only models of TMOUD may include the marginalization of those who feel able to use the telephone to receive care even if data-heavy mobile apps or video calls are unaffordable or unappealing.

Another example is the inclusion criterion for accepting patients into a TMOUD program. Clinical inclusion criteria may typically incorporate measures of stability such as housing status, criminal activity, polysubstance use, and the existence of concurrent mental health issues [57]. A TMOUD service that excludes people who are unstably housed or have comorbid health conditions may systematically exclude the most vulnerable among its catchment population [57]. Furthermore, in the United States, people categorized as “unstable” based on these criteria tend to belong to people of color populations, leading to exclusion along racial lines [57].

#### Stakeholder Engagement

It is important to engage with stakeholders at all levels of service delivery. Nevertheless, depending on the standard model of care in a particular jurisdiction, not all stakeholder groups may play a critical role in TMOUD implementation. Most services will need to engage effectively with leadership and management stakeholders, such as clinical, organizational, operational, and nursing managers, and prescribers, such as medical directors, physicians, physician assistants, and nurse practitioners. However, not all services will have the same community pharmacy arrangements depending on the availability of pharmacy outlets in the region, the existence of locally negotiated service-level agreements, and whether electronic prescriptions for controlled drugs are permissible within that jurisdiction.

Other differences across jurisdictions may be the level of access to behavioral health personnel such as social workers, clinical psychologists, mental health therapists, substance use counselors, and case managers. In addition, some aspects of care may be delivered by organizations without the relevant data-sharing arrangements and access to shared records, for example, community outreach workers or employees of third-sector housing, harm reduction, or advocacy services. Different agencies may have different levels of clinical and IT administrator and program coordinator support. Finally, patients, particularly those not already known to the service, may be at a significant disadvantage as their needs may be entirely invisible to TMOUD service implementors.

What this effectively means is that, after identifying key stakeholders, a protracted and challenging period of engagement and negotiation may be necessary to enable them to play their roles in TMOUD delivery. Among the most challenging arrangements are those involving community pharmacies and electronic prescriptions. Unlike in the United States, community pharmacies in the United Kingdom may dispense methadone and buprenorphine following the instructions of the prescriber, including the provision of observed dosing [89]. Although this is a significant advantage to MOUD provision in the United Kingdom, the regulatory aspects and reimbursement structure for electronic prescribing and dispensing are currently ambiguous and pose a barrier to TMOUD.

#### Service Design

##### Inclusion and Exclusion Criteria

A nuanced approach to identifying suitability for TMOUD is to use criteria stratified according to the risk level and stage of treatment. This should be in addition to regulatory requirements and existing service-level policies. Considerations may include (1) clinical circumstances such as comorbidities, particularly severe mental health diagnoses, pregnancy, polypharmacy, polysubstance use, previous treatment experiences, and frailty, and (2) social and personal circumstances such as the remoteness of the person’s location, poor transport access, local COVID-19 restrictions, access to technology, risk of travel versus the risk of overdose, and whether the person lives alone or in supported accommodation. A standard template for triage or suitability for TMOUD should be developed to direct referrals appropriately, and alternative methods of assessment should be identified for people excluded from TMOUD. Multimedia Appendix 7 provides examples of exclusion and inclusion criteria for fully web-based and hybrid TMOUD.

##### Models of TMOUD Delivery

As the practice of TMOUD has grown throughout the pandemic, several models of care surrounding this intervention have emerged. One of the reviews carried out within this project [57] categorized 4 overlapping models of TMOUD delivery elaborated on in Textbox 2. These were TMOUD to provide an inclusion health focus, facilitate transitions through care, meet complex care needs, and increase service resiliency and quality. Services intending to adopt TMOUD may find that their existing or intended care model matches some elements of each of these categories or is in alignment with one specific category. Defining the key principles of care delivery underpinning TMOUD will have several implications, including the selection of quality and outcome measures of importance. This will be elaborated on in a later section.

**Models of telemedicine-delivered medication for opioid use disorder (TMOUD).**

**TMOUD model (TM) 1.0: inclusion health focus**

- Definition: the model is focused on reaching marginalized, easily ignored groups at the intersections of multiple forms of exclusion, including race, homelessness, criminal justice involvement, and forced displacement.
- Key aspects of care include outreach through street medicine, mobile clinics, health care facilitators, harm reduction workers, telemedicine facilitators, and multicomponent care.
- TM 1.1: places of safety. Care is brought to the patient where they are and feel safe.
- TM 1.2: trauma informed. The service is guided by trauma-informed principles and culturally appropriate, peer-supported care.
- TM 1.3: low threshold. Provides low-threshold, no–wrong door, low-barrier entry into treatment.
- TM 1.4: social determinants of health. The service is sensitive to social determinants of health, including digital exclusion, and mitigates them.

**TM 2.0: transitions through care**

- Definition: the model is focused on promoting the patient’s social stability and continuity of care across jurisdictional boundaries and points of transition where overdose risk may be magnified.
- Key aspects of care include bridge clinics and shared care arrangements among specialist, primary care, and multidisciplinary services such as joint opioid use disorder and perinatal services.
- TM 2.1: patient journey mapping. Comprises the identification of points of high risk of patient attrition, for example, leaving prison; discharge from hospital, emergency department, or treatment service; and becoming homeless or relocating.
- TM 2.2: process mapping and risk mitigation. Removes barriers to accessing a medication for opioid use disorder (MOUD) prescription, clinical review, or MOUD supply through a dispensing pharmacy.
- TM 2.3: chronic condition management. Incorporates principles of managing a chronic relapsing or remitting condition where longitudinal continuity of care reduces the risk of adverse health outcomes.
- TM 2.4: multiple episodes of care. Comprises recognizing, accepting, and allowing for multiple episodes of care across the condition.

**TM 3.0: complexity of care needs**

- Definition: the model is designed around multiple unmet care needs, including housing; legal and income support; treatment of comorbid physical and mental health conditions; and harm reduction interventions to prevent blood-borne virus transmission, soft tissue infections, and overdose deaths.
- Key aspects of care include care navigators or medical case managers to coordinate interactions with health services, navigate complex bureaucratic systems, create individualized care plans, and support patients in meeting their goals.
- TM 3.1 comprises multidisciplinary and multisectoral care incorporating social workers, counselors, nurses, primary care physicians, case managers, legal advisors, and outreach workers.
- TM 3.2 comprises the flexibility to tailor interventions or programs to different needs, for example, services for people with HIV or Hepatitis C, inclusion health primary care, or street medicine services.
- TM 3.3 comprises education and empowerment of patients through peers or culturally appropriate workers to facilitate self-advocacy.

**TM 4.0: increasing service resilience and quality**

- Definition: the model is designed around an existing service’s needs to fill resource or geographic gaps; remove administrative or clinical burden; introduce cost efficiencies; or increase flexibility to innovate, change, adapt, or survive.
- Key aspects of care are determined at the organizational level with defined goals, criteria, boundaries, and processes, and there may be limited opportunities for service user involvement in its design and delivery.
- TM 4.1: internally or externally sourced. TMOUD pathways and expertise are developed internally or brought in from an external vendor.
- TM 4.2: predefined intake criteria. The TMOUD service has specific intake criteria. Where a TMOUD vendor is used, intake may rely on shared care protocols with the host organization to ensure that an appropriate screening process has taken place before referral.
- TM 4.3: coproduced clinical pathways. Where a vendor is used, service delivery pathways will need to be coproduced with the host organization to fit with the organization’s strategic and pragmatic aims in introducing TMOUD.

Note: Models from Teck et al [57].

##### Agreed Upon Modes of TMOUD Delivery: Overview

A total of 4 modes of TMOUD delivery have been identified in the literature. One of the 4 modes exclusively involved web-based contact, whereas the other 3 were hybrid forms of TMOUD in which some in-person contact was involved. The hub-home model is frequently described in the literature and involves the clinician performing the TMOUD visit with the person in their home [36]. An initial screening assessment and triage may have taken place via telephone before prescriber contact [65], and specific arrangements may have had to be put in place for identity confirmation and urine drug testing before the consultation [36,65,90]. The triadic hub-home model is a derivative of the more common hub-home model, in which a health care assistant or bridge worker attends the person’s home to facilitate the TMOUD consultation. This is particularly helpful when the person may be disabled, struggles to use technology, or has additional needs such as the administration of a depot medication. Examples were not readily found in the literature; however, some key informants had experience using this model.

The hub-spoke model involves the person accessing technology to enable TMOUD in a setting outside their home. The triadic version of this model involved the presence of a health care assistant or bridge worker to facilitate the TMOUD consultation. The flexibility offered by the hub-spoke model meant that it was offered in a broad range of settings, including prisons, needle and syringe provision services, and rural primary care [62,63,91].

One of our stakeholder organizations—an advocacy group for families affected by drugs and alcohol—highlighted the lack of examples of family-inclusive practices in the literature on TMOUD modes of delivery. For example, within the context of the hub-spoke model, family members who often perform informal caring roles may be well suited to being TMOUD facilitators, ensuring that the person can go on the web, providing corroborative history, advocating for their family member’s needs, or collecting medications from a dispensing pharmacy.

##### Agreed Upon Modes of TMOUD Delivery: Agreement on Who Should Carry Out the TMOUD Consultation

The choice of prescriber to perform TMOUD consultations depends on a variety of factors. By choice of prescriber, we mean the profession to which they belong (eg, pharmacy, nursing, or medicine); their level of seniority, experience, and training; and the degree of autonomy permitted to them by their respective organizations and governing bodies. For example, the General Medical Council in the United Kingdom highlighted specific areas of concern and risk when delivering telemedicine [92]. Issues must have been identified and addressed before there can be an expectation of a UK-registered physician to start providing TMOUD consultations, and different professions may have different requirements.

An example of a medical student conducting the initial assessment of a person accessing TMOUD, followed by a discussion and consultation with a fully qualified physician, was identified [93], highlighting the value for the next generation of clinicians preparing for what the future of addiction care may look like. Indeed, the incorporation of digital health competencies into the medical school curriculum is becoming increasingly important worldwide [94]. Another consideration is that clinical seniority and experience in providing MOUD may not translate easily to high-quality care via TMOUD. Indeed, there is evidence that some clinicians experienced difficulties in the rapid transition from in-person consultations to telemedicine at the pandemic onset [36]. Understanding TMOUD as a digital health intervention with specific skills and competencies is currently an underresearched area.

##### Clinic Coordination and Standard Operating Procedures

Key issues in this area included the ways in which people contact the service to access TMOUD and how the service would accommodate people with transient lifestyles, limited access to technology, and difficulties keeping their handsets safe or charged. Examples of street outreach services actively engaging homeless populations [35] and needle and syringe provision services acting as locations for TMOUD [91] may help overcome some of these issues. Key informants who provided frontline support services highlighted the increased demand for alternate nonverbal means of communication, such as web chat, email, or SMS text messaging, particularly in cases in which people may be tentatively exploring the idea of engaging with services. The need to involve support workers, families, or telemedicine facilitators outside the main provider’s governance structure may require additional guidance or memoranda of understanding, allowing staff to work in more flexible yet defensible ways when making appointments or coordinating other aspects of care. Furthermore, standard lone workers or physical consultation risk assessments and safety policies will need to be modified for web-based environments. Multimedia Appendix 8 details other important factors to consider before TMOUD consultations may begin. We summarized in visual form the steps involved in an actual TMOUD consultation, adapting the work by the Welsh National Video Consultation Service Toolkit [95], in Multimedia Appendix 9.

##### Quality Standards and Monitoring

Quality standards for TMOUD provision will relate closely to the type of care that should be offered in response to the gaps and needs of the current system and, where relevant, statutory benchmarking, for example, the MAT standards outlined in Textbox 1. In Table 1, we outline specific service-level measures that relate to the Scottish MAT standards and show how they map to models of TMOUD derived from the international evidence base. Using the measures in Table 1 will not only support ongoing evaluation and service improvement but also enable benchmarking against international examples of TMOUD models of care.

**Table 1:** Medication-assisted treatment (MAT) standards 1 to 5 mapped against telemedicine-delivered medication for opioid use disorder (TMOUD) models of care.

Scottish MAT standards and what to measure			Mapping against TMOUD models of care		Implications for TMOUD quality
**Standard 1: same-day access**					
	Time from first contact with any partner in the multiagency partnership within an episode of care to the commencement of MOUD^a^	TM^b^ 1.0: inclusion health focus TM 1.1: places of safety TM 1.3: low threshold TM 3.1: multidisciplinary and multisectoral care		Same-day access is made possible by low thresholds for treatment initiation (TM 1.3). Multidisciplinary and multisectoral care (TM 3.1) increases opportunity for people to be seen where they feel safe (TM 1.1).	
	Number of people started on MOUD	TM 1.3: low threshold TM 4.0: service resilience and quality		Higher numbers in treatment are a measure of treatment threshold (TM 1.3) and may be an outcome measure required by a service adopting TMOUD (TM 4.0).	
**Standard 2: choice**					
	The number of people in treatment taking methadone or sublingual buprenorphine at the appropriate dose and with opportunities to change their choice	TM 1.2: trauma informed TM 3.3: education and empowerment TM 4.0: service resilience and quality TM 4.3: coproduced clinical pathways		Choice in treatment is a principle of trauma-informed care (TM 1.2), and people with opioid use disorder may need to be empowered to demand these choices (TM 3.3). Services need to have time and resources (TM 4.0) and appropriate clinical pathways (TM 4.3) to respond to demand.	
	The number of people in treatment receiving injectable buprenorphine	TM 1.0: inclusion health focus TM 3.3: education and empowerment TM 4.0: service resilience and quality		Some groups may be excluded from being offered injectable buprenorphine (TM 1.0) or may not have knowledge of it (TM 4.0). Services must adapt to introduce injectable buprenorphine, and adopting TMOUD may increase the capacity to do so (TM 4.0).	
	The number of people receiving HAT^c^	TM 1.0: inclusion health focus TM 3.0: complexity of care needs TM 4.0: service resilience and quality		HAT is a relatively novel intervention, potentially benefiting marginalized populations (TM 1.0) with complex care needs (TM 3.0). It is a resource-heavy intervention, and TMOUD may be used to free up capacity to deliver it (TM 4.0).	
**Standard 3: assertive outreach and anticipatory care**					
	Duration from when first identified as at risk to initial contact and assessment; number of people followed up with and for whom an initial assessment is performed	TM 2.0: transition through care TM 2.1: patient journey mapping TM 2.2: process mapping and risk mitigation		This measure may provide empirical data to test service understandings of the patient journey (TM 2.1) and processes that may increase or decrease risk (TM 2.2) during transitions in care (TM 2.0).	
	Proportion of people by age, gender, and race identified as at risk by source of risk event (service that identified and actioned the risk); identification and outcome of the intervention	TM 1.0: inclusion health focus TM 1.1: places of safety TM 2.0: transition through care TM 2.2: process mapping and risk mitigation		Disaggregation of data by age, gender, race, and other characteristics measures the extent to which inclusivity is improved (TM 1.0), what services are favored by specific groups (TM 1.1), and whether risk mitigation (TM 2.2) during care transitions (TM 2.0) reaches all affected groups equally.	
**Standard 4: harm reduction**					
	Proportion of MOUD services offering BBV^d^ testing and vaccination, naloxone and overdose awareness, wound care, assessment of injecting risk and injecting equipment provision, and virtual supervised injecting services	TM 3.0: complexity of care needs TM 3.2: flexibility to tailor interventions TM 4.0: service resilience and quality TM 4.3: coproduced clinical pathways		This measure quantifies the extent to which TMOUD services the complexity of the needs of people with opioid use disorder (TM 3.0) and the extent to which it can innovate or adapt to address gaps (TM 3.2). Furthermore, services may introduce telemedicine (TM 4.0) and coproduce pathways to integrate harm reduction and MOUD provision.	
**Standard 5: retention**					
	Attrition rate: number of people currently on MOUD treatment and number of people discharged within a set time by setting, age, gender, and race	TM 2.0: transition through care TM 2.1: patient journey mapping TM 2.2: process mapping and risk mitigation TM 4.0: service resilience and quality TM 4.3: coproduced clinical pathways		This directly measures the service’s ability to support continuity through transitions in care (TM 2.0) and provides empirical data to test mapping and risk mitigation (TM 2.1 and 2.2). TMOUD may be adopted to improve retention in treatment (TM 4.0) through specific clinical pathways (TM 4.3).	
	Reason for discharge (eg, planned or unplanned)	TM 3.0: complexity of care needs TM 3.2: flexibility to tailor interventions TM 4.0: service resilience and quality TM 4.2: predefined intake criteria		Services designed to meet complex needs (TM 3.0) develop interventions to match these needs (TM 3.2) and, theoretically, will have low unplanned discharges. Services introducing TMOUD to increase efficiency (TM 4.0) may set high-threshold intake criteria (TM 4.2), which excludes some groups.	

^a^MOUD: medication for opioid use disorder.

^b^TM: TMOUD model.

^c^HAT: heroin-assisted treatment.

^d^BBV: blood-borne virus.

#### Key Process Domains

Understanding how MOUD is defined and offered within a service is essential to understanding not only where the introduction of telemedicine would be most beneficial but also what the potential risks and CRM strategies should be. Several national and international guidance documents have set out how the core aspects and quality standards of MOUD services should be provided and demonstrated, including those from the United Kingdom [67], Scotland [4], the United States [96], and the World Health Organization [97]. With reference to these documents, we divided the processes involved in MOUD into those performed before the commencement of medication (accessing the service, enrollment, diagnosis of OUD, and treatment planning) and processes associated with medication commencement (induction, medication titration, substance use stabilization, and regular medication and clinical reviews). Textboxes 3 and 4 describe these processes and the associated risks involved in designing a TMOUD service. When introducing a pilot TMOUD project, clinicians and planners may decide to offer telemedicine for specific domains while also ensuring that arrangements are in place for other nontelemedicine domains. It is also critical for providers to evaluate the extent to which they can accommodate people who do not wish to have telemedicine consultations, particularly outside of pandemic or public health–mandated situations. These processes are often illustrated in the form of a process map [98], and an example of this is provided in Multimedia Appendix 10.

**Processes before the commencement of medication for opioid use disorder (MOUD) and considerations when transitioning to telemedicine.**

 

**Entry points**

- Discovery: How will people find out about the telemedicine-delivered MOUD (TMOUD) service? Examples: word of mouth, internet, social media, referrals from other agencies, advertisements or flyers, or outreach or in-reach services.
- Accessibility: What will the service opening times be? By being on the web or through the telephone, are drop-in options no longer possible? What about out-of-hours access? Will there be a free phone number? Will this free phone number be integrated alongside other service numbers, for example, for unscheduled or emergency care? Will the service be by referral only?
- Safety and inclusivity: How are the principles of a psychologically informed, welcoming, trustworthy, private, transparent, and respectful service maintained through telemedicine? How are peer support; family involvement; collaborative working; and sex-, language-, and culturally appropriate services provided through telemedicine?
- Associated risks in planning for access:
  Easily ignored or marginalized groups may not access the relevant information on TMOUD services in a timely way or have the skills to act on them.
  Fear of stigmatizing or disrespectful attitudes may discourage people from attending a TMOUD consult.
  Rigid appointment times may not suit this group, making the availability of a drop-in option important.
  People with no fixed address will not receive postal appointments and may struggle to retain the same contact number throughout their treatment.
  Although telemedicine has been described as having the potential to improve access to MOUD, the reality needs to be closely monitored as delays are associated with increased mortality and morbidity among people with opioid use disorder (OUD).

**Enrollment**

- Identity: confirming the identity of people experiencing homelessness and other itinerant or marginalized groups can be problematic yet essential when providing controlled drugs as medication. In addition, registration with a primary care service and the ability to check that the person is not already accessing these medications through another service are critical and may be more difficult via a fully web-based service. In some settings, eligibility to access the service and consent forms may need to be verified in person.
- Past medical history: timely access to medical records; the person’s MOUD treatment history; recent investigations including liver function tests, drug tests, and electrocardiograms; information on comorbidities; recent hospital admissions; other prescribed medication; allergies; and contraindications are essential for safe MOUD induction.
- Additional needs such as blood-borne virus (BBV) testing; sterile injecting equipment access; take-home naloxone supplies; primary care; mental health, financial, housing, and nutritional support; advocacy; and a dispensing pharmacy are necessary components of care to be arranged.
- Risks:
  Delays in the enrollment processes may delay safe prescribing, increasing the risk of withdrawals and contributing to increased mortality and morbidity risks.
  Opioid withdrawals are independently associated with receptive syringe sharing and nonfatal overdose.
  Failure to conduct the necessary checks may result in double prescribing or harms such as overdose from unsafe prescribing. The safety standards of the service may be brought into question, resulting in sanctions.
  MOUD is most effective in preventing HIV and hepatitis C transmission if provided in the context of a robust sterile injecting equipment supply.

**Diagnosis**

- Assessment: information that contributes to a robust assessment includes the substances used, preferred route of administration (eg, sniffing, smoking, or injection), daily use (amount and cost), desired effect (eg, up, down, or feeling normal), tolerance, withdrawal symptoms, age at first use, strengths or skills, social support, criminal justice involvement, drug mixing, infection risk, nonfatal overdose frequency, psychiatric symptoms, previous treatment experiences, and whether the person meets validated criteria for OUD diagnosis.
- Examination: includes assessment of pulse, evidence of tremor or agitation, flushing, sweating, pupil size, yawning, irritability, mental state, injecting sites, and jaundice.
- Investigations or standardized tools: drug testing, pregnancy testing, liver function tests, electrocardiograms, and BBV near-person testing form part of standard good practice in-person MOUD provision. Many services are also required to collect a minimum data set to meet statutory or insurance provider obligations.
- Risks:
  A diagnosis of OUD is an absolute prerequisite to initiate MOUD such as methadone and buprenorphine.
  An overestimation of a person’s level of tolerance to opioids may result in an overdose.
  Where the necessary information is collated, it is possible to make a diagnosis of dependence and make a prescribing decision at the first appointment.
  Individuals already well known to the service can often be safely reassessed and restarted on treatment rapidly, particularly if their information is relatively recent.
  Arbitrary attendance to multiple appointments before initiating treatment increases risks of harm to the person.

**Treatment planning**

- Reviewing potential harms and risks both of entering treatment and of the person’s current situation: this includes the risk of overdose and death, infection, social isolation, acquisitive crime and incarceration, drug debts, violence, exploitation, homelessness, family breakdown, and increased suicidality.
- Exploring the person’s priorities and perspectives: there is an ethical and legal obligation upon care providers to ensure that the person’s values and priorities are understood and considered in clinical decision-making. For example, the person may wish to find a way to continue to use drugs safely, reduce the harms of their ongoing drug use, reduce their use to controllable levels, or stop entirely. They may wish to work toward abstinence and move on to rehabilitation services or seek sufficient stability to pursue other forms of personal development.
- Supporting an informed treatment choice: the mechanism of action of each MOUD option (typically methadone or buprenorphine) needs to be understood by the person, including the risks and benefits. There may be pharmacological prerequisites to be understood, such as needing a period without using before initiating buprenorphine. The clinician is typically able to make recommendations only after a discussion on the potential risk-benefit and the person’s preference.
- Risks:
  Credibility, trust, and rapport are required for treatment recommendations to be accepted.
  A mismatch between the clinician’s and the individual’s outcome goals can result in disengagement from services.
  Telemedicine may hamper the degree of communication required for a person to fully understand the information provided. Both clinicians and individuals may feel pressured into concluding the consultation quickly owing to discomfort with the web-based platform.

**Processes during and following the commencement of medication for opioid use disorder (MOUD) and considerations when transitioning to telemedicine.**

**Induction**

- Safety: the identity of the person taking the medication needs to be confirmed, and a clinician able to determine their state at the time of induction (intoxication, withdrawals, or neutral) is required. A breath alcohol level may be necessary. Immediate action or follow-up arrangements may be needed if a risk of an overdose or harm is noted.
- Medication-specific issues: buprenorphine has a lower risk of respiratory depression and overdose in someone who has an opioid tolerance, and unobserved home induction may be possible. Conventionally, this requires definite withdrawal symptoms to avoid the possibility of induced withdrawals. As a full opioid agonist, methadone carries additional risk, and there are greater risks of harm with unobserved dosing at this stage of treatment.
- The first dose of treatment: Where will dispensing occur? Who will supervise the dose? Is the person required to be seen again after receiving their first dose? What is the feasibility of offering injectable formulations of MOUD via telemedicine?
- Risks:
  Both the clinician and the dispensing pharmacy will need to confirm the person’s identity.
  With polysubstance use, for example, alcohol or benzodiazepine codependence, the status of the person at the point of induction may be difficult to determine via voice or video-based telemedicine.
  Clear plans need to be in place for when a person attending a telemedicine-delivered MOUD (TMOUD) consultation is clearly unwell or intoxicated. These plans need to be feasible and timely, for example, ensuring emergency services or that take-home naloxone administration can occur to prevent an overdose.

**Titration**

- Attendance: titrating the dose of MOUD to an adequate level may require several encounters with a prescriber (daily, twice a week, or weekly). This is a critical phase as the person may feel the need to continue to use until the treatment takes the withdrawals away. Nonattendance to a review appointment is difficult to manage in both a telemedicine or in-person context. A plan needs to be in place to manage nonattendance, including how safeguarding or check-ins will be performed. Other considerations include a management plan for losses to follow-up and strategies to increase retention in treatment.
- Support: outreach support, TMOUD facilitators, appointment reminders, tracing service if appointments are missed, peer or psychosocial support, and ongoing harm reduction interventions such as take-home naloxone provision can significantly improve engagement and safety of the titration phase and compensate for the lack of in-person clinical contact.
- Dosing-related issues: frequency of dose increases and consideration of how these are managed with the dispensing pharmacy are critical. Will the person need to come to the service to pick up a prescription? Will the pharmacy accept electronic prescriptions? How will missed doses or intoxication observed by the pharmacist be managed?
- Risks:
  The risk of death increases at the beginning and end of MOUD treatment.
  Methadone initiation requires prescribing at subtherapeutic doses and subsequent titration to a therapeutic dose over a few weeks, resulting in risks with ongoing drug use and death, making the first 4 weeks of treatment risky.
  Supervised consumption is used to reduce the risk of overdose and diversion and is associated with a reduction in illicit heroin and alcohol use but may be associated with decreased retention in treatment.
  Mortality risk at treatment onset is lower among those initiated on buprenorphine than methadone; however, retention may be better with methadone.
  Currently, experience with TMOUD has been predominantly with buprenorphine rather than methadone.

**Stabilization**

- Comorbidities: a period of stabilization may provide opportunities for people engaging with MOUD to begin to manage coexisting conditions such as HIV, hepatitis C, and tuberculosis treatment.
- Managing other substances: polysubstance use with alcohol and illicit substances or prescription opioids limits the benefits gained from MOUD. The period of stability through MOUD commencement may support the person to seek help with these other substances.
- Psychosocial interventions: contingency management, cognitive behavioral therapy, relapse prevention, dialectical behavior therapy, group drug counseling, mutual aid including 12-step and Specific, Measurable, Attainable, Relevant, and Time-Bound goals, employment support, education and training, upskilling, benefit maximization, legal support, advocacy, compassion-focused therapy, and mindfulness have all been successfully delivered via a telemedicine platform and should be considered in the design of TMOUD services.
- Risks:
  People who have stabilized in their substance use may become more aware of previously hidden psychiatric symptoms and will need timely access to mental health services. Anticipating and offering this within a telemedicine service is important though not always feasible.
  A service transitioning to telemedicine needs to provide avenues to access other services critical to the person’s ongoing welfare to maximize the benefits of MOUD.

**Medication reviews**

- Adapting to changing circumstances: the TMOUD service will need to respond to the changing health circumstances of individuals, for example, the onset of cardiovascular or respiratory disease as the person ages. An individual’s needs may change through their treatment journey, and flexibility in dispensing arrangements may be required, which should be incorporated into the TMOUD service policies. The TMOUD service will need to have provision for prescribing adjustments, for example, where individuals have palliative or chronic pain needs.
- Managing relapses: a clear plan for how relapses are managed must be in place, including dosing adjustments and harm reduction interventions. Is the person at greater risk of relapsing without the appropriate support or would it be undetected if engaging with a TMOUD rather than an in-person service?
- Moving forward: Does the service provide or link with other services? Are there opportunities to access long-acting MOUD formulations, which reduce the person’s need to attend a dispensing pharmacy regularly?
- Risks:
  A telemedicine service may not provide the usual touch points available in an in-person service, which may trigger professional curiosity or opportunities for exploring topics unrelated to MOUD provision alone.
  Consequently, the TMOUD service may become less relevant to the individuals’ circumstances; however, the need for arrangements for change, transfer, or discharge may not be anticipated.

#### Iterative Cycles of Evaluation and Improvement

##### A Quality Improvement Focus

Existing guidance on telemedicine service transition during and beyond the COVID-19 pandemic has recommended a quality improvement approach to TMOUD implementation [62,69] echoed by several key informants. This may include the development of a theory of change, a phased implementation approach, and iterative testing to develop and refine the processes informed by feedback from stakeholders [99]. Another approach identified in the literature is the use of formal implementation frameworks to evaluate TMOUD projects. For example, the Consolidated Framework for Implementation Research was used in one study as a conceptual guide for the systematic assessment of barriers and facilitators in different multilevel implementation contexts to influence TMOUD effectiveness [62]. Using the Consolidated Framework for Implementation Research, Brunet et al [62] extended the transferability of their evaluation findings to other services in diverse settings.

##### Evaluation

In addition to service-level quality measures identified in Table 1, validated tools to evaluate TMOUD more generally may be useful. Several evaluation tools were identified in the literature, including the Telemedicine Satisfaction Questionnaire [100] evaluating the usability of the telemedicine service [101] and the Telemedicine Service Maturity Model [102,103] to evaluate the implementation of telemedicine services. One key study reviewed several existing tools to develop a survey-based measurement tool for TMOUD satisfaction among people who use drugs receiving this treatment in rural areas [104].

## Discussion

### Principal Findings

We have reported on a multistakeholder, process-focused implementation model for TMOUD. This model is intended to provide clear step-by-step guidance on TMOUD implementation informed by evidence from the peer-reviewed literature as well as internationally derived case study expertise. Although this work is admittedly focused on the Scottish context, the use of the SHIFT-Evidence framework to underpin model development, along with the international evidence base, may increase its relevance to a wider, international audience. Through stakeholder engagement and consultation, we tried to mitigate the perception of a top-down imposition on how TMOUD should be delivered or implemented. Our consultation efforts resulted in the capture and inclusion of a wide range of knowledge, perspectives, experiences, and opinions. A limitation is the lack of a specific mechanism to form a consensus on what is critical to the model, for example, through a survey-based Delphi process. We have secured funding to field-test this TMOUD implementation model in Scotland, after which we will seek formal consensus on its validity in other settings.

Another limitation of our study was a reliance on a US-dominated evidence base, which >70% of TMOUD research currently comes from [48]. Experience in the United States tends to involve the provision of buprenorphine via telemedicine, whereas services in the United Kingdom tend to prescribe methadone more often than buprenorphine. This may mean that UK services need to develop a robust homegrown evidence base to provide methadone via telemedicine to fully benefit from TMOUD implementation. Furthermore, the evidence base grew substantially from the time this work began to the point of submission for peer review. Built into the design of this project were iterative and flexible scoping reviews and ongoing consultation with the community of practice derived from the case study identification stage.

There is currently a gap in evidence-based risk mitigation strategies when delivering TMOUD, identified in the process-mapping activity described previously. Furthermore, there is a need to develop technological approaches to improve the role of telemedicine platforms to act as a diagnostic platform as well as a medium for consultations when delivering TMOUD. Proposals for this include adapting smartphone-based pupillometry techniques to assess the degree of withdrawal from opioids [105] and a process for web-based point-of-care drug testing [106]. Although it is recognized that frontline practitioners and people who use drugs should be partners in the design, implementation, and evaluation of TMOUD [48], there is little evidence on how to engage these groups in a meaningful and impactful way.

### Conclusions

In conclusion, we have produced a process-driven TMOUD implementation model intended to bridge gaps in knowledge at the local level while also providing flexibility to respond to local needs and contexts. A critical next step will be to field-test the model and seek international consensus on its validity across different settings.

## Appendix

Multimedia Appendix 1 Applying the 12 Successful Healthcare Improvements From Translation of Evidence Into Practice rules to the study design.

Multimedia Appendix 2 Logic model.

Multimedia Appendix 3 Web-based stakeholder engagement event topic list.

Multimedia Appendix 4 Draft implementation model (PDF form).

Multimedia Appendix 5 Screenshots of the web-based platform used to consult on the implementation model.

Multimedia Appendix 6 Diversity of stakeholders and key informants in the consultation phase.

Multimedia Appendix 7 Examples of inclusion and exclusion criteria for telemedicine-delivered medication for opioid use disorder.

Multimedia Appendix 8 Clinic coordination and administration.

Multimedia Appendix 9 Visual guide to conducting a telemedicine-delivered medication for opioid use disorder consultation.

Multimedia Appendix 10 Telemedicine-delivered medication for opioid use disorder process map.

## Abbreviations

ADP: Alcohol and Drug Partnership
CRM: clinical risk management
DRD: drug-related death
MAT: medication-assisted treatment
MOUD: medication for opioid use disorder
OUD: opioid use disorder
SHIFT-Evidence: Successful Healthcare Improvements From Translation of Evidence Into Practice
TMOUD: telemedicine-delivered medication for opioid use disorder

## References

[1] McAuley A, Fraser R, Glancy M, Yeung A, Jones HE, Vickerman P, Fraser H, Allen L, McDonald SA, Stone J, Liddell D, Barnsdale L, Priyadarshi S, Markoulidakis A, Hickman M, Hutchinson SJ (2023). Mortality among individuals prescribed opioid-agonist therapy in Scotland, UK, 2011-20: a national retrospective cohort study. Lancet Public Health.

[2] Baumgartner R (2021). Precision medicine and digital phenotyping: digital medicine's way from more data to better health. Big Data Soc.

[3] (2022). Changing lives: our final report. Scottish Drug Deaths Taskforce.

[4] (2021). Medication Assisted Treatment (MAT) standards for Scotland access, choice, support. Scottish Government.

[5] Santo T Jr, Clark B, Hickman M, Grebely J, Campbell G, Sordo L, Chen A, Tran LT, Bharat C, Padmanathan P, Cousins G, Dupouy J, Kelty E, Muga R, Nosyk B, Min J, Pavarin R, Farrell M, Degenhardt L (2021). Association of opioid agonist treatment with all-cause mortality and specific causes of death among people with opioid dependence: a systematic review and meta-analysis. JAMA Psychiatry.

[6] Volkow ND, Frieden TR, Hyde PS, Cha SS (2014). Medication-assisted therapies--tackling the opioid-overdose epidemic. N Engl J Med.

[7] Winograd RP, Presnall N, Stringfellow E, Wood C, Horn P, Duello A, Green L, Rudder T (2019). The case for a medication first approach to the treatment of opioid use disorder. Am J Drug Alcohol Abuse.

[8] Marchand K, Beaumont S, Westfall J, MacDonald S, Harrison S, Marsh DC, Schechter MT, Oviedo-Joekes E (2019). Conceptualizing patient-centered care for substance use disorder treatment: findings from a systematic scoping review. Subst Abuse Treat Prev Policy.

[9] Donroe JH, Tetrault JM (2018). Narrowing the treatment gap in managing opioid use disorder. CMAJ.

[10] Barry CL, McGinty EE, Pescosolido BA, Goldman HH (2014). Stigma, discrimination, treatment effectiveness, and policy: public views about drug addiction and mental illness. Psychiatr Serv.

[11] McGinty EE, Stone EM, Kennedy-Hendricks A, Bachhuber MA, Barry CL (2020). Medication for opioid use disorder: a national survey of primary care physicians. Ann Intern Med.

[12] Eisenberg MD, McCourt A, Stuart EA, Rutkow L, Tormohlen KN, Fingerhood MI, Quintero L, White SA, McGinty EE (2021). Studying how state health services delivery policies can mitigate the effects of disasters on drug addiction treatment and overdose: protocol for a mixed-methods study. PLoS One.

[13] Miclette MA, Leff JA, Cuan I, Samet JH, Saloner B, Mendell G, Bao Y, Ashburn MA, Bachhuber MA, Schackman BR, Polsky DE, Meisel ZF (2018). Closing the gaps in opioid use disorder research, policy and practice: conference proceedings. Addict Sci Clin Pract.

[14] Mojtabai R, Mauro C, Wall MM, Barry CL, Olfson M (2019). Medication treatment for opioid use disorders in substance use treatment facilities. Health Aff (Millwood).

[15] Stringer KL, Langdon KJ, McKenzie M, Brockmann B, Marotta P (2021). Leveraging COVID-19 to sustain regulatory flexibility in the treatment of opioid use disorder. J Subst Abuse Treat.

[16] Feder KA, Krawczyk N, Saloner B (2017). Medication-assisted treatment for adolescents in specialty treatment for opioid use disorder. J Adolesc Health.

[17] Frazer Z, McConnell K, Jansson LM (2019). Treatment for substance use disorders in pregnant women: motivators and barriers. Drug Alcohol Depend.

[18] Krans EE, Kim JY, James AE 3rd, Kelley D, Jarlenski MP (2019). Medication-assisted treatment use among pregnant women with opioid use disorder. Obstet Gynecol.

[19] Salvador J, Bhatt S, Fowler R, Ritz J, James R, Jacobsohn V, Brakey HR, Sussman AL (2019). Engagement with project ECHO to increase medication-assisted treatment in rural primary care. Psychiatr Serv.

[20] Tyndall M, Dodd Z (2020). How structural violence, prohibition, and stigma have paralyzed north American responses to opioid overdose. AMA J Ethics.

[21] Allden K, Murakami N, Maung C (2015). Trauma and Recovery on War's Border: A Guide for Global Health Workers.

[22] Kane JC, Ventevogel P, Spiegel P, Bass JK, van Ommeren M, Tol WA (2014). Mental, neurological, and substance use problems among refugees in primary health care: analysis of the Health Information System in 90 refugee camps. BMC Med.

[23] Miler JA, Carver H, Masterton W, Parkes T, Maden M, Jones L, Sumnall H (2021). What treatment and services are effective for people who are homeless and use drugs? A systematic 'review of reviews'. PLoS One.

[24] Dickie E, Clusker T, McCormick D (2021). Medication assisted treatment MAT standards for Scotland access, choice, support interim report. Scottish Drug Deaths Taskforce.

[25] The Scottish Drug Deaths Task Force evidence paper. The Scottish Drug Deaths Task Force.

[26] Eibl JK, Morin K, Leinonen E, Marsh DC (2017). The state of opioid agonist therapy in Canada 20 years after federal oversight. Can J Psychiatry.

[27] Eibl JK, Gauthier G, Pellegrini D, Daiter J, Varenbut M, Hogenbirk JC, Marsh DC (2017). The effectiveness of telemedicine-delivered opioid agonist therapy in a supervised clinical setting. Drug Alcohol Depend.

[28] Huskamp HA, Busch AB, Souza J, Uscher-Pines L, Rose S, Wilcock A, Landon BE, Mehrotra A (2018). How is telemedicine being used in opioid and other substance use disorder treatment?. Health Aff (Millwood).

[29] Molfenter T, Boyle M, Holloway D, Zwick J (2015). Trends in telemedicine use in addiction treatment. Addict Sci Clin Pract.

[30] Wang L, Weiss J, Ryan EB, Waldman J, Rubin S, Griffin JL (2021). Telemedicine increases access to buprenorphine initiation during the COVID-19 pandemic. J Subst Abuse Treat.

[31] Weintraub E, Greenblatt AD, Chang J, Himelhoch S, Welsh C (2018). Expanding access to buprenorphine treatment in rural areas with the use of telemedicine. Am J Addict.

[32] Krawczyk N, Fawole A, Yang J, Tofighi B (2021). Early innovations in opioid use disorder treatment and harm reduction during the COVID-19 pandemic: a scoping review. Addict Sci Clin Pract.

[33] Cantor J, McBain RK, Kofner A, Hanson R, Stein BD, Yu H (2022). Telehealth adoption by mental health and substance use disorder treatment facilities in the COVID-19 pandemic. Psychiatr Serv.

[34] Tofighi B, McNeely J, Walzer D, Fansiwala K, Demner A, Chaudhury CS, Subudhi I, Schatz D, Reed T, Krawczyk N (2022). A telemedicine buprenorphine clinic to serve New York City: initial evaluation of the NYC public hospital system's initiative to expand treatment access during the COVID-19 pandemic. J Addict Med.

[35] Tringale R, Subica AM (2021). COVID-19 innovations in medication for addiction treatment at a Skid Row syringe exchange. J Subst Abuse Treat.

[36] Uscher-Pines L, Sousa J, Raja P, Mehrotra A, Barnett M, Huskamp HA (2020). Treatment of opioid use disorder during COVID-19: experiences of clinicians transitioning to telemedicine. J Subst Abuse Treat.

[37] Watson DP, Swartz JA, Robison-Taylor L, Mackesy-Amiti ME, Erwin K, Gastala N, Jimenez AD, Staton MD, Messmer S (2021). Syringe service program-based telemedicine linkage to opioid use disorder treatment: protocol for the STAMINA randomized control trial. BMC Public Health.

[38] Ghosh A, Mahintamani T, Pillai RR, Subodh BN, Mattoo SK, Basu D (2021). Telemedicine-assisted stepwise approach of service delivery for substance use disorders in India. Asian J Psychiatr.

[39] (2023). Why trust digital health? Understanding the perspectives of communities affected by BBVs/STIs and social stigma. Research Gate.

[40] Kho J, Gillespie N, Martin-Khan M (2020). A systematic scoping review of change management practices used for telemedicine service implementations. BMC Health Serv Res.

[41] Lin LA, Casteel D, Shigekawa E, Weyrich MS, Roby DH, McMenamin SB (2019). Telemedicine-delivered treatment interventions for substance use disorders: a systematic review. J Subst Abuse Treat.

[42] Nabitz U, van Den Brink W, Jansen P (2005). Using concept mapping to design an indicator framework for addiction treatment centres. Int J Qual Health Care.

[43] Teck JT, Baldacchino AM, el-Guebaly N, Carrà G, Galanter M, Baldacchino AM (2021). COVID-19 and substance use disorders: syndemic responses to a global pandemic. Textbook of Addiction Treatment: International Perspectives.

[44] Trujols J, Garijo I, Siñol N, del Pozo J, Portella MJ, Pérez de los Cobos J (2012). Patient satisfaction with methadone maintenance treatment: the relevance of participation in treatment and social functioning. Drug Alcohol Depend.

[45] Walters SM, Perlman DC, Guarino H, Mateu-Gelabert P, Frank D (2022). Lessons from the first wave of COVID-19 for improved medications for opioid use disorder (MOUD) treatment: benefits of easier access, extended take homes, and new delivery modalities. Subst Use Misuse.

[46] (2022). National benchmarking report on implementation of the medication assisted treatment (MAT) standards. Public Health Scotland.

[47] Update on medication assisted treatment standards. Health and social care news, Scottish Government.

[48] Teck JT, Zlatkute G, Perez A, Dritschel H, Ghosh A, Potenza MN, Ambekar A, Ekhtiari H, Stein D, Khazaal Y, Arunogiri S, Torrens M, Ferri M, Galea-Singer S, Baldacchino A (2023). Key implementation factors in telemedicine-delivered medications for opioid use disorder: a scoping review informed by normalisation process theory. The Lancet Psychiatry.

[49] Scottish G (2012). A national telehealth and telecare delivery plan for Scotland to 2015. The Scottish Government.

[50] Wherton J, Greenhalgh T Evaluation of the attend anywhere/near me video consulting service in Scotland, 2019-20. Scottish Government Edinburgh.

[51] Wherton J, Greenhalgh T, Shaw SE (2021). Expanding video consultation services at pace and scale in Scotland during the COVID-19 pandemic: national mixed methods case study. J Med Internet Res.

[52] Wherton J, Greenhalgh T (2020). Evaluation of the near me video consulting service in Scotland during COVID-19. Scottish Government.

[53] Joudrey PJ, Adams ZM, Bach P, Van Buren S, Chaiton JA, Ehrenfeld L, Guerra ME, Gleeson B, Kimmel SD, Medley A, Mekideche W, Paquet M, Sung M, Wang M, You Kheang RO, Zhang J, Wang EA, Edelman EJ (2021). Methadone access for opioid use disorder during the COVID-19 pandemic within the United States and Canada. JAMA Netw Open.

[54] Reed JE, Howe C, Doyle C, Bell D (2018). Simple rules for evidence translation in complex systems: a qualitative study. BMC Med.

[55] McLaughlin JA, Jordan GB (1999). Logic models: a tool for telling your programs performance story. Eval Program Plann.

[56] Mishuris RG, Palmisano J, McCullagh L, Hess R, Feldstein DA, Smith PD, McGinn T, Mann DM (2019). Using normalisation process theory to understand workflow implications of decision support implementation across diverse primary care settings. BMJ Health Care Inform.

[57] Tay Wee Teck J, Butner JL, Baldacchino A (2023). Understanding the use of telemedicine across different opioid use disorder treatment models: a scoping review. J Telemed Telecare.

[58] Gottlieb M, Haas MR, Daniel M, Chan TM (2021). The scoping review: a flexible, inclusive, and iterative approach to knowledge synthesis. AEM Educ Train.

[59] Gordon I (2021). Seminar delivering medication assited treatment for addictions via telehealth. School of Medicine, University of St Andrews.

[60] (2020). Coronavirus resilience planning: use of near me videoconsulting- organisation. Technology Enabled Care Scotland.

[61] (2021). Digital citizen delivery plan 2021/2022. Technology Enabled Care Scotland.

[62] Brunet N, Moore DT, Lendvai Wischik D, Mattocks KM, Rosen MI (2022). Increasing buprenorphine access for veterans with opioid use disorder in rural clinics using telemedicine. Subst Abus.

[63] Duncan A, Sanders N, Schiff M, Winkelman TN (2021). Adaptations to jail-based buprenorphine treatment during the COVID-19 pandemic. J Subst Abuse Treat.

[64] Tofighi B, Isaacs N, Byrnes-Enoch H, Lakew R, Lee JD, Berry C, Schatz D (2019). Expanding treatment for opioid use disorder in publicly funded primary care clinics: exploratory evaluation of the NYC health + hospitals buprenorphine ECHO program. J Subst Abuse Treat.

[65] Crowley D, Delargy I (2020). A national model of remote care for assessing and providing opioid agonist treatment during the COVID-19 pandemic: a report. Harm Reduct J.

[66] Fiacco L, Pearson BL, Jordan R (2021). Telemedicine works for treating substance use disorder: the STAR clinic experience during COVID-19. J Subst Abuse Treat.

[67] (2017). Clinical guidelines on drug misuse and dependence update. Independent Expert Working Group.

[68] Lingam HA, Caudill RL (2019). Teleprescribing controlled substances: flowchart analysis of the Ryan Haight act and state telemedicine laws. J Technol Behav Sci.

[69] Weintraub E, Seneviratne C, Anane J, Coble K, Magidson J, Kattakuzhy S, Greenblatt A, Welsh C, Pappas A, Ross TL, Belcher AM (2021). Mobile telemedicine for buprenorphine treatment in rural populations with opioid use disorder. JAMA Netw Open.

[70] Lofwall MR, Walsh SL (2014). A review of buprenorphine diversion and misuse: the current evidence base and experiences from around the world. J Addict Med.

[71] Nosyk B, Slaunwhite A, Urbanoski K, Hongdilokkul N, Palis H, Lock K, Min JE, Zhao B, Card KG, Barker B, Meilleur L, Burmeister C, Thomson E, Beck-McGreevy P, Pauly B (2021). Evaluation of risk mitigation measures for people with substance use disorders to address the dual public health crises of COVID-19 and overdose in British Columbia: a mixed-method study protocol. BMJ Open.

[72] Day N, Wass M, Smith K (2022). Virtual opioid agonist treatment: Alberta's virtual opioid dependency program and outcomes. Addict Sci Clin Pract.

[73] Durand L, Keenan E, Boland F, Harnedy N, Delargy Í, Scully M, Mayock P, Ebbitt W, Vázquez MO, Corrigan N, Killeen N, Pate M, Byrne P, Cousins G (2022). Consensus recommendations for opioid agonist treatment following the introduction of emergency clinical guidelines in Ireland during the COVID-19 pandemic: a national Delphi study. Int J Drug Policy.

[74] Hew A, Arunogiri S, Lubman DI (2021). Challenges in delivering telemedicine to vulnerable populations: experiences of an addiction medical service during COVID-19. Med J Aust.

[75] (2020). FAQs: provision of methadone and buprenorphine for the treatment of opioid use disorder in the COVID-19 emergency. Mental Health Services Administration.

[76] Hser YI, Ober AJ, Dopp AR, Lin C, Osterhage KP, Clingan SE, Mooney LJ, Curtis ME, Marsch LA, McLeman B, Hichborn E, Lester LS, Baldwin LM, Liu Y, Jacobs P, Saxon AJ (2021). Is telemedicine the answer to rural expansion of medication treatment for opioid use disorder? Early experiences in the feasibility study phase of a national drug abuse treatment clinical trials network trial. Addict Sci Clin Pract.

[77] (2019). NHS in Scotland 2019. Audit Scotland.

[78] Black C (2021). Review of drugs part two: prevention, treatment, and recovery. Government of UK.

[79] Briner M, Kessler O, Pfeiffer Y, Wehner T, Manser T (2010). Assessing hospitals' clinical risk management: development of a monitoring instrument. BMC Health Serv Res.

[80] Oesterle TS, Kolla B, Risma CJ, Breitinger SA, Rakocevic DB, Loukianova LL, Hall-Flavin DK, Gentry MT, Rummans TA, Chauhan M, Gold MS (2020). Substance use disorders and telehealth in the COVID-19 pandemic era: a new outlook. Mayo Clin Proc.

[81] Chan B, Bougatsos C, Priest K, McCarty D, Grusing S, Chou R (2022). Opioid treatment programs, telemedicine and COVID-19: a scoping review. Subst Abus.

[82] Holmes H, Burgess G (2022). Digital exclusion and poverty in the UK: how structural inequality shapes experiences of getting online. Digit Geogr Soc.

[83] (2022). Connecting Scotland: phase 1 evaluation. Scottish Government.

[84] Hadi D, Hannah C, Catriona M, Tessa P, Joe S, Graeme S (2022). Digital inclusion to prevent drug related deaths: literature review. Drugs Research Network Scotland.

[85] Dorney-Smith S, Burridge S, Bell J, Ellis J, Snowball L Digital health inclusion for people who have experienced homelessness- is this a realistic aspiration?. Pathway.

[86] Wunsch C, Wightman R, Pratty C, Jacka B, Hallowell BD, Clark S, Davis CS, Samuels EA (2023). Thirty-day treatment continuation after audio-only buprenorphine telehealth initiation. J Addict Med.

[87] Yeo EJ, Kralles H, Sternberg D, McCullough D, Nadanasabesan A, Mayo R, Akselrod H, Catalanotti J (2021). Implementing a low-threshold audio-only telehealth model for medication-assisted treatment of opioid use disorder at a community-based non-profit organization in Washington, D.C. Harm Reduct J.

[88] Kennedy AJ, George JS, Rossetti G, Brown CO, Ragins K, Dadiomov D, Trotzky-Sirr R, Sanchez G, Llamas H, Hurley B (2023). Providing low-barrier addiction treatment via a telemedicine consultation service during the COVID-19 pandemic in Los Angeles, county: an assessment 1 year later. J Addict Med.

[89] Brandee I, Frances M, Sheri D UK pharmacies offer sense of normalcy for methadone patients. PEW Charitable Trust.

[90] Guille C, Simpson A, Douglas E, Boyars L, Cristaldi K, McElligott J, Johnson D, Brady K (2020). Treatment of opioid use disorder in pregnant women via telemedicine: a nonrandomized controlled trial. JAMA Netw Open.

[91] Lambdin BH, Kan D, Kral AH (2022). Improving equity and access to buprenorphine treatment through telemedicine at syringe services programs. Subst Abuse Treat Prev Policy.

[92] (2022). Regulatory approaches to telemedicine. Europe Economics. General Medical Council.

[93] Castillo M, Conte B, Hinkes S, Mathew M, Na CJ, Norindr A, Serota DP, Forrest DW, Deshpande AR, Bartholomew TS, Tookes HE (2020). Implementation of a medical student-run telemedicine program for medications for opioid use disorder during the COVID-19 pandemic. Harm Reduct J.

[94] Khurana MP, Raaschou-Pedersen DE, Kurtzhals J, Bardram JE, Ostrowski SR, Bundgaard JS (2022). Digital health competencies in medical school education: a scoping review and Delphi method study. BMC Med Educ.

[95] Using video consultations in secondary and hospital care: a toolkit for clinicians. DigitalHealth.

[96] (2021). TIP 63: medications for opioid use disorder for healthcare and addiction professionals, policymakers, patients, and families. Substance Abuse and Mental Health Services Administration.

[97] (2020). International standards for the treatment of drug use disorders: revised edition incorporating results of field-testing. World Health Organization.

[98] Antonacci G, Lennox L, Barlow J, Evans L, Reed J (2021). Process mapping in healthcare: a systematic review. BMC Health Serv Res.

[99] (2020). An evaluation of our rapid implementation support for NHS Near Me 23 March-30 June 2020. Healthcare Improvement Scotland.

[100] Yip MP, Chang AM, Chan J, MacKenzie AE (2003). Development of the telemedicine satisfaction questionnaire to evaluate patient satisfaction with telemedicine: a preliminary study. J Telemed Telecare.

[101] Parmanto B, Lewis AN Jr, Graham KM, Bertolet MH (2016). Development of the Telehealth Usability Questionnaire (TUQ). Int J Telerehabil.

[102] Otto L, Whitehouse D, Schlieter H (2019). On the road to telemedicine maturity: a systematic review and classification of telemedicine maturity models. BLED 2019 Proceedings.

[103] van Dyk L, Fortuin J, Schutte C (2012). A maturity model for telemedicine implementation. Proceedings of the 4th International Conference on eHealth, Telemedicine, and Social Medicine.

[104] Cole TO, Robinson D, Kelley-Freeman A, Gandhi D, Greenblatt AD, Weintraub E, Belcher AM (2020). Patient satisfaction with medications for opioid use disorder treatment via telemedicine: brief literature review and development of a new assessment. Front Public Health.

[105] McGrath LB, Eaton J, Abecassis IJ, Maxin A, Kelly C, Chesnut RM, Levitt MR (2022). Mobile smartphone-based digital pupillometry curves in the diagnosis of traumatic brain injury. Front Neurosci.

[106] (2022). Virtual MOUD treatment: virtual point-of-care toxicology testing to accompany virtual medication assisted treatment for opioid use disorder. US National Library of Medicine.

