# Supplementary material for: Developing a Theoretically Informed Implementation Model for Telemedicine-Delivered Medication for Opioid Use Disorder: Qualitative Study With Key Informants
Source: JMIR Ment Health. 2023 Oct 18;10:e47186. doi: 10.2196/47186 (PMC10620637; doi:10.2196/47186)
Supplement: Multimedia Appendix 1 [file mental_v10i1e47186_app1.docx]

###### Multimedia Appendix 1: Applying the twelve Successful Healthcare Improvement From Translating Evidence in complex systems (SHIFT-Evidence) rules to this study.

| **Strategic principles** | **Simple rules** | **Application to this study**^1^ |
| --- | --- | --- |
| Act scientifically and pragmatically | Understand the problem and opportunities. | 1.10 Review of Scottish telemedicine and tele-health policy, strategy and implementation guidance documents and scale-up evaluation reports  1.12 Understanding the addiction treatment context and the impact of the MAT standards.  2.51 Identification of case studies of TMOUD implementation  3.10 Stakeholder mapping  3.11 Stakeholder engagement event |
|  | Identify, test and iteratively develop potential solutions. | 2.10 Mapping out best practice for MOUD delivery.  2.20 Interpreting the evidence base against theoretical frameworks such as normalisation process theory to facilitate transferability to different contexts.  2.21 Understanding the different TMOUD models and contexts of care to facilitate translation of the evidence base |
|  | Assess whether improvement is achieved, capture and share learning. | 1.30 The scope of the implementation model encompasses this.  1.31 Defining TMOUD.  1.33 Implementation and delivery processes.  1.34 Reflexive monitoring and improvement. |
|  | Invest in continual improvement. | 1.34 The implementation model will outline how reflexive monitoring should occur to enable iterative improvements in both the implementation model and the TMOUD service itself. |
| Embrace complexity | Understand practices and processes of care. | 2.20 and 2.21 focussed on understanding practices and processes of care, with further key informant input in 5.0. |
|  | Understand types and sources of variation. | 2.20 Key implementation factors in telemedicine-delivered medications for opioid use disorder  2.21 TMOUD models of care: inclusion health, transitions through care, complexity of care needs, service resilience |
|  | Identify systemic issues as learning emerges, where additional support is needed and how it may be obtained. | 1.31 Understanding of how the intervention is different to standard practice.  1.33 Process Mapping, risk-assessment process and mitigation action.  5.11 Strategic alignment, leadership, linking with key decision-makers.  6.50 Funding secured to pilot the model. |
|  | Seek political, strategic and financial alignment and target resources to improvements with the greatest impact. | 1.33 Understanding practices and processes of care.  2.21 Understanding the different models and contexts of care of TMOUD.  1.34 Quality and standards and monitoring. |
|  | Actively engage those responsible for and affected by change | 3.10 Stakeholder mapping.  3.11 Stakeholder engagement event to generate awareness and interest in the potential of TMOUD.  5.0 Key informant (stakeholder) consultation. |
| Engage and empower | Facilitate dialogue to develop a common understanding of the system, build trust, relationships and generate active reflection. | 5.0 Key informant (stakeholder) consultation incorporating an asynchronous virtual platform (5.10), anonymous commenting on the draft document (5.10) and a final round table discussion (5.11) |
|  | Build a culture of willingness to learn and freedom to act. | 1.30 Undertaking a participatory approach in developing this model to avoid top-down imposition of specific practices.  1.32 Building relationships: Providing guidance on the identification of and engagement with stakeholders, to build the necessary relationships in implementing TMOUD.  1.34 Outlining how reflexive monitoring should occur to enable for iterative improvements in both the implementation model and the TMOUD service itself. |
|  | Provide headroom, resources, training, and support. | 1.34 Outlining how reflexive monitoring should occur to enable iterative improvements in both the implementation model and the TMOUD service itself.  6.50 Funding has been secured to pilot the implementation model in Scotland. Currently identifying service delivery partner. (Ongoing work) |
|  | Invest in continual improvement. | 1.34 Outlining how reflexive monitoring should occur to enable iterative improvements in both the implementation model and the TMOUD service itself.  2.0 Identification of evidence to inform the implementation model: Iterative systematic review in response to knowledge gaps as there are identified. |

^1^ The numbering in this column refers to specific numbered items in the logic model shown in Multimedia Appendix 2.
