# Supplementary figures and images for: Developing a Theoretically Informed Implementation Model for Telemedicine-Delivered Medication for Opioid Use Disorder: Qualitative Study With Key Informants
Source: JMIR Ment Health. 2023 Oct 18;10:e47186. doi: 10.2196/47186 (PMC10620637; doi:10.2196/47186)

Multimedia Appendix 2: **Logic model of the six-step research design**

**
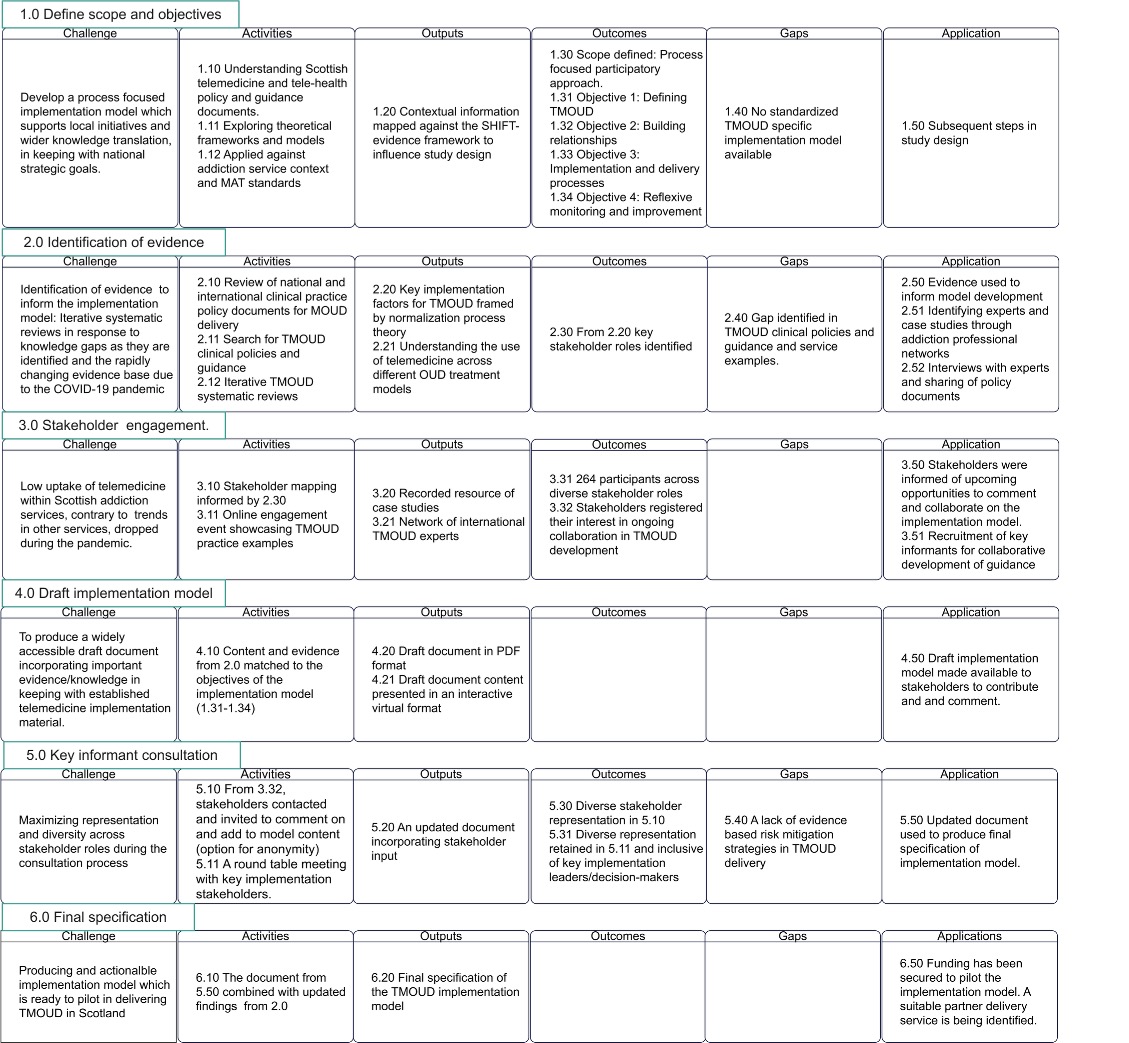
**

Supplement: Multimedia Appendix 2 [file mental_v10i1e47186_app2.docx]

Multimedia Appendix 9. A visual guide to TMOUD delivery


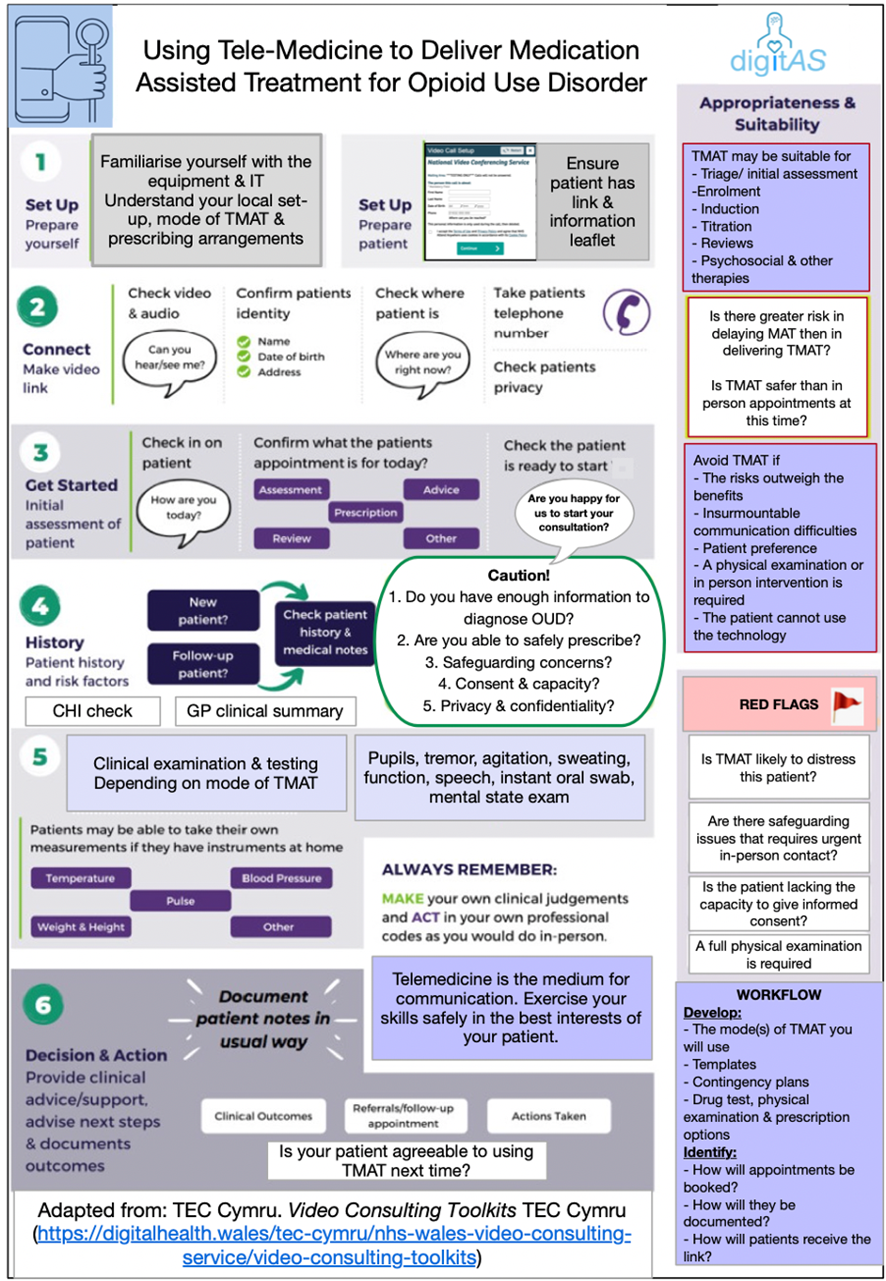

Supplement: Multimedia Appendix 9 [file mental_v10i1e47186_app9.docx]
