# Supplementary material for: Developing a Theoretically Informed Implementation Model for Telemedicine-Delivered Medication for Opioid Use Disorder: Qualitative Study With Key Informants
Source: JMIR Ment Health. 2023 Oct 18;10:e47186. doi: 10.2196/47186 (PMC10620637; doi:10.2196/47186)
Supplement: Multimedia Appendix 3 [file mental_v10i1e47186_app3.docx]

Table 2. Case studies and topics presented at the TMOUD engagement virtual seminar

| **Location** | **Service or topic** | **Key learning points** |
| --- | --- | --- |
| Province-wide, Alberta. Canada | The Virtual Opioid Dependency Program (VODP) | - Unique challenge raised by fentanyl-crisis with drug-related deaths and working across a vast geography - Designed to be a fully virtual MOUD delivery service - Multiple direct access and referral options - Public service providing methadone and buprenorphine, including same day initiation - Provincewide, but using local community infrastructure including pharmacies and lab facilities |
| St Louis, USA | Assisted Recovery Centers of America | - Unique challenge posed by COVID-19 - Private practice delivering virtual, limited contact and street outreach, including at new venues to better support where patients have health/transportation/mobility issues - Use of alternative access options (e.g. text message, email) - Use of digitalised internal pathways to improve patient flow |
| Calgary, Alberta, Canada | Delivering non-opioid medication assisted treatment via telehealth | - Unique challenge posed by COVID-19: moved to complete virtual and now combination of virtual/in-person - Operating from a community health centre, accepting walk-ins/self-referrals/advance phone appointments - Low threshold for pharmacological and psychosocial treatment and developed home alcohol detox protocol - Rapid same day assessment and 1-3 day access to a prescriber |
| Nationwide, Republic of Ireland | Telehealth delivery of opioid substitute treatment/addiction medicines during the COVID-19 pandemic | - Unique challenges posed by COVID-19 led to increased use of phone/video consultations: enabled more people to access treatment but concerns about possible privacy/digital poverty issues - Service delivery predominantly via GPs, also central treatment centres/satellite clinics - Provides methadone and buprenorphine where individuals are centrally registered - Change in legislation allowed prescriptions to be emailed to community pharmacies - Reduced drug screening not associated with significant problems - Secure email system improves communication between healthcare professionals |
| British Colombia, Canada | Setting up virtual medically assisted treatment delivery during a pandemic | - Unique challenge of the opioid-crisis, COVID-19 and large geographical spread with large numbers needing treatment and limited physician coverage - Virtual clinics offered during the week, weekends and holiday periods - After drug test and brief history, virtual appointment (within 2 hours) with a physician, prescription faxed to pharmacy to enable same day access to medication - Highlights importance of internal infrastructure and functional technology - Challenges in transferring care to community, non-virtual physicians |
| Scotland, England and Wales | Pharmacy implications of medication assisted treatment through telehealth | - Outlined the importance of considering the role of pharmacy, with particular reference to service access, assessment, prescribing and medicines supply with associated governance issues - Challenges include digital poverty, rurality, provision of drug tests, accessing relevant heath records, lack of e-prescribing systems and legal requirements |
| Yorkshire and Humber, England | Telemedicine in addictions: feasibility RCT | - Hub and spoke outreach access, with same day assessment, drug test and prescription production - Compared virtual and face to face consult service delivery for methadone and buprenorphine - No major changes in engagement/satisfaction rates/clinical outcomes - Challenges with technology |
| Nationwide, Scotland | A systems approach to telehealth implementation | - Unique challenge posed by COVID-19 - Unscheduled care services across large geography - Virtual delivery supported by investment in hardware, clinical systems/processes/governance and staff training/support - Highlighted need for IT glitches and buy-in from system leaders/clinicians |
