# Supplementary material for: Developing a Theoretically Informed Implementation Model for Telemedicine-Delivered Medication for Opioid Use Disorder: Qualitative Study With Key Informants
Source: JMIR Ment Health. 2023 Oct 18;10:e47186. doi: 10.2196/47186 (PMC10620637; doi:10.2196/47186)

Good Practice Guidance for the Design & Delivery of a Telemedicine Medication Assisted Treatment (TMAT) Service for Opioid Use Disorder (OUD)

Table of Contents

INTRODUCTION 2

Purpose of this guide **2**

METHODOLOGY 4

STEP-BY-STEP GUIDE 5

1. Setting up your TMAT service **5**

2. Risk, safeguarding and ethical considerations **9**
   Risk-assessment process. 9
   Process Mapping 9

3. A guide to conducting the TMAT consultation **13**
   Modes of TMAT 13
   14
   The TMAT Consultation 15

Evaluation component **16**

CONCLUSIONS 17

REFERENCES 19

DRAFT PLEASE DO NOT DISTRIBUTE

Introduction

Scotland has the highest per capita Drug Related Death (DRD) rate in Europe, with opioids implicated in 86% of cases (Office for National Statistics, 2019). In July 2019, the Scottish Government set up the Drug Deaths Task Force (DDTF) (Drug Deaths Task Force, 2020) to stem this rising trend which has seen the DRD rate double since 2008. A key DDTF priority has been to support service innovations which improve ease of access to and available options of Medication Assisted Treatment (MAT) for People Who Use Drugs (PWUD). Innovative, flexible and responsive MAT has become even more important during the COVID-19 pandemic not least as people who use opioids and other drugs have heightened health and social risks increasing their vulnerability to poor outcomes.

SCOTTISH DRUG DEATHS TASKFORCE

IMPROVING SCOTLAND'S HEALTH

Rights, Respect and Recovery

1,264

#StopTheDeaths

1,264 people tragically lost their lives to a preventable drug overdose in Scotland last year. The statistics, published by National Records of Scotland, show the toll is 6% higher than 2018, and the highest since records began in 1996. Scotland's drug death rate is now 3.5 times higher than the rest of the UK and is higher than that reported for any other EU country.

Medication Assisted Treatment (MAT)

defined as

'the use of medication, such as opioids, together with psychological and social support, in the treatment and care of individuals who experience problems with their drug use'.

Aim: *improve quality and consistency of care across the country.*

Medication Assisted Treatment subgroup

DRAFT PLEASE DO NOT DISTRIBUTE

Purpose of this guide

We see this guide as a resource for the implementation of TMAT in keeping with the proposed national MAT standards. This guide does not review the evidence for the MAT standards themselves which have been reported elsewhere (Johns et al., 2020). Figure 1. maps out the relationship between the MAT standards and the various aspects of TMAT delivery. The knowledge and literature around the implementation of specific healthcare interventions are not always readily accessible in the public domain. Specifically, when it comes to technology based interventions, or interventions in response to crises, the published literature becomes rapidly outdated (Mohr et al., 2015). We have therefore taken the Digital Design for Addiction Services (Colley & Marttila, 2017, p. 295-8) approach which is described in more detail below.

Figure 1. The Medication Assisted Treatment (MAT) standards mapped against Telemedicine Medication Assisted Treatment (TMAT) delivery.

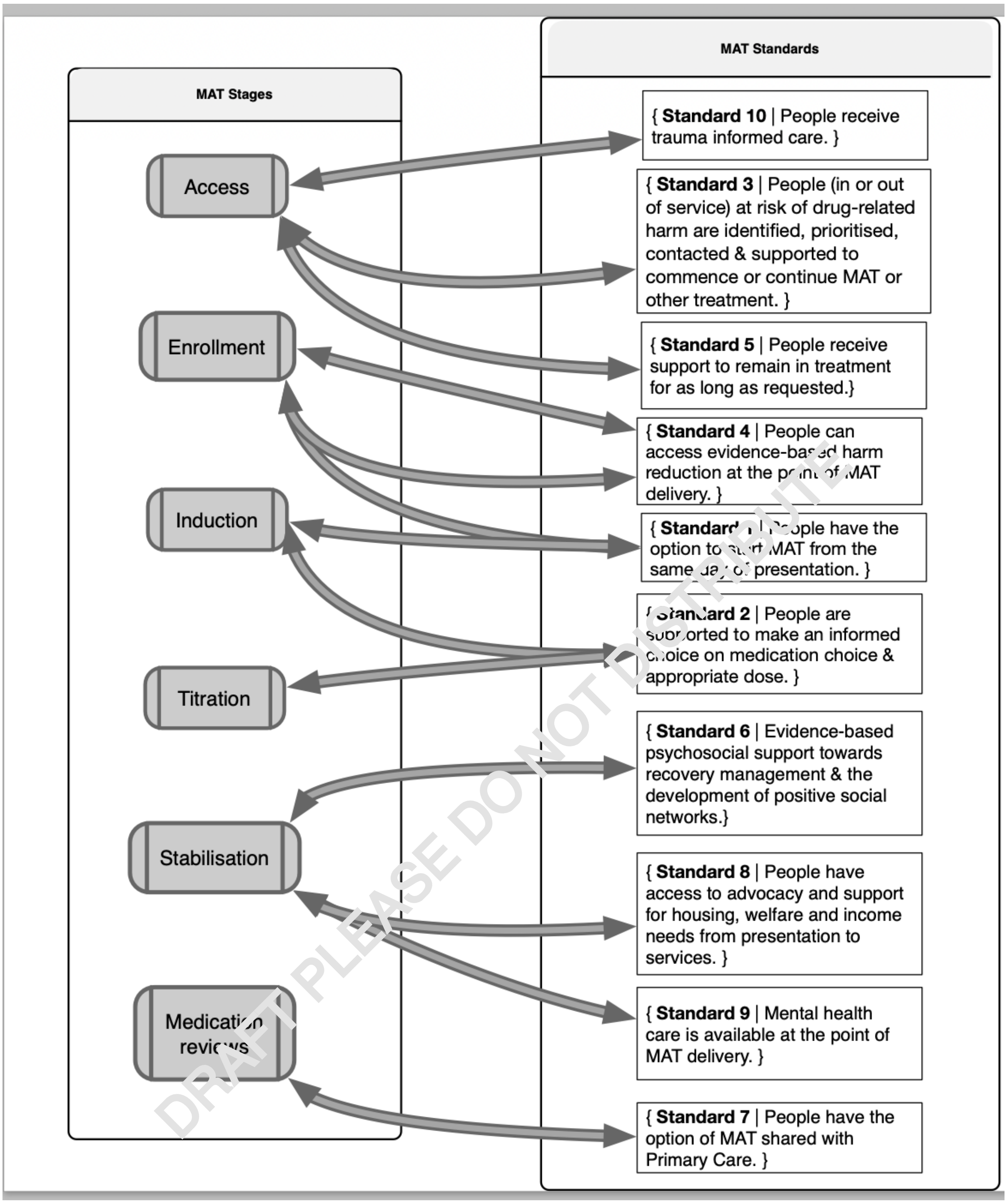

DRAFT PLEASE DO NOT DISTRIBUTE

Comments

[redacted] This is a really confusing slide esp where a new concept to the reader e.g. may not know what trauma informed care actually means. Also the arrows and where standards are linked in doesn't really fit e.g. TIA, access to MH, housing support etc should actually be something that occurs throughout care provision

Methodology

In this project we have committed to a design philosophy based on person centredness, visual and inclusive communication, collaboration & co-creation and continuous (iterative) improvement. Figure 2. describes the process we have undertaken to align with these philosophical underpinnings.

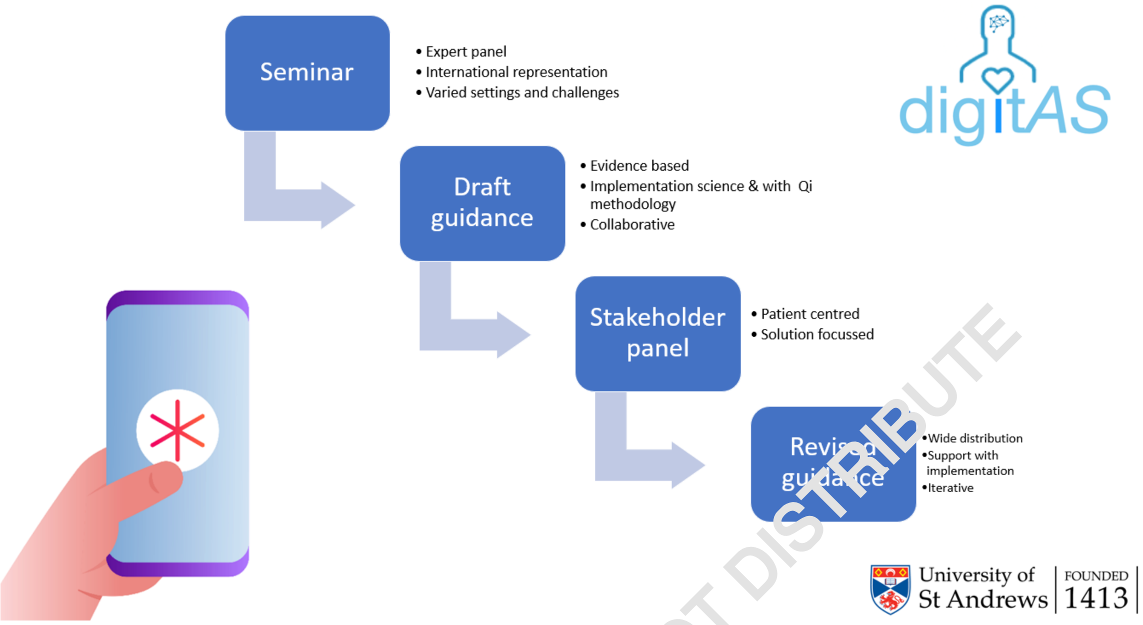

Figure 2. The TMAT guidelines co-production process.

DRAFT PLEASE DO NOT DISTRIBUTE

We have completed stage one, a seminar which capitalised on the wealth of experience gained by colleagues during the pandemic and earlier, in comparable international and national health systems with similar drug related death rates and service challenges. The seminar presentations and recordings are available on the digitAS website: http://med.st-andrews.ac.uk/digitas/.

Following on from the seminar which serves as a shared body of knowledge, we are launching this draft guidance. The guidance is intended to support the process of identifying the best point of patient contact to introduce telemedicine consultations. It also goes through the process of setting up Telemedicine Medication Assisted Treatment (TMAT) in existing systems of care, a guide to risk, safeguarding and ethical guidelines and a guide to the consultation process itself. In addition to the expertise derived from our panel, we have also conducted a rapid literature review and we refer extensively to the visual step-by-step guide for clinicians using video consultations in mental health services (Johns et al., 2020) and a series of toolkits produced by Technology Enabled Care (TEC) Cymru (TEC Cymru, 2021).

Comments

[redacted]: Would you want to highlight the global nature of this e.g. US, NZ etc.?

[redacted] QI quality improvement (in reference to Figure 2)

Step-by-step guide

This guide describes Telemedicine Medication Assisted Treatment (TMAT) delivery in three ways. The first section describes a set up process and considerations around TMAT delivery in an existing healthcare workflow. The second section discusses the ethical and legal principles to be considered in TMAT delivery. The final section discusses the actual TMAT consultation. We use tables and diagrams extensively to make this guide more easily adapted to local needs and to make information more readily available when required.

1. Setting up your TMAT service

Table 1. TMAT service design

Process	Considerations
**1. Identify a lead clinician**	Strategic view and understanding of local strengths, infrastructure and risks.<br><br>Lead discussions and make decisions on best set-up to meet local circumstances and needs.<br><br>Decide on appropriate management structure and delegate tasks and accountability appropriately.<br><br>Evaluate and improve service in response to patient and clinical need.
**2. Identify stages of Medication Assisted Treatment (MAT) most appropriate for your new clinic**	Will telemedicine be used for all or only certain stages of MAT?<br><br>(Stages of MAT: Access, Enrolment, Induction, Titration, Stabilisation, Reviews)<br><br>Will it be piloted in one of the stages and then expanded to others as experience grows?<br><br>What stage(s) is/are immediately amenable to telemedicine?
**3. Define & agree clinical criteria for Telemedicine Medication Assisted Treatment (TMAT) use**	These criteria should incorporate patient need, special considerations around choice of Medication Assisted Treatment (MAT), a risk-benefit evaluation, opportunity costs. Clinical criteria may be stratified according to risk level, and MAT stage.<br><br>Considerations include *clinical circumstances* such as co-morbidities, particularly severe mental health diagnoses, pregnancy, poly-pharmacy, poly-substance use, previous treatment experiences and frailty, as well as *social and personal circumstances* such as remote location, poor transport access, local Covid restrictions, access to technology, risk of travel versus risk of overdose, and whether the person lives alone or in supported accommodation.
**4. Develop a template for triage/suitability for TMAT**	This template can help direct referrals from a single point of contact to the TMAT service if appropriate
**5. Agree upon mode(s) of TMAT delivery**	Four modes are described in more detail below. In brief: Mode 1. **Hub-home**: Clinician connects from clinic to patient at home.<br><br>Mode 2. **Dyadic hub-spoke**: Clinician in specialist hub centre connects to patient in remote spoke health spoke or care site without additional staff member present (e.g. in an unstaffed kiosk).

DRAFT PLEASE DO NOT DISTRIBUTE

	Mode 3. <u>**Triadic hub-spoke**</u>: Clinician in specialist 'hub' centre connects to patient in remote 'spoke' healthcare site (for example, a community pharmacy, GP practice) with another healthcare worker present (for example, nurse, pharmacist, healthcare support worker)<br><br>Mode 4. **Triadic hub-home**: Clinician in specialist 'hub' centre connects to patient at home with another healthcare worker present (for example, nurse, GP, healthcare support worker).
**6. Healthcare worker safety**	Ensure that the lone worker and risk assessment guidance is adapted accordingly.
**7. Who will conduct the TMAT consultation?**	Will it be delivered by any independent prescriber?<br><br>Will it be piloted initially with a more senior clinician?<br><br>Will it be piloted by a senior clinician alongside another prescriber so that learning and experience is shared?
**8. How will TMAT be delivered?**	NHS Near Me is the agreed national platform in Scotland and therefore all clinician to patient video calling should occur on Near Me. Other platforms do not necessarily have the information governance or security measures in place. However, some services have used Skype for business, and some services in North America have used encrypted ZOOM calls. Some services also offer drop in video clinics. Additionally, some private companies in the UK have bespoke mobile platforms.
**9. Identify how TMAT links and information will be sent to patient e.g. SMS, email, verbally**	Appointment links and information may be sent via a Short Message Service (SMS), text or email, or the link may be embedded on a webpage that patient is directed to.
**10. Identify how TMAT appointments will be booked and documented**	Identify person(s) in charge of making the TMAT appointments. This person(s) should counter check that contact information such as email addresses or mobile numbers for SMS are correct to avoid the link for the appointment going to the wrong person.<br>How much in advance can TMAT appointments be booked? This is particularly relevant in populations which may not have consistent mobile phone numbers or addresses.
**11. Appointment system**	How will appointment slots be offered, documented and given to the delivering clinician? Does the system being used to deliver TMAT integrate well with existing appointment systems?
**12. Set up the clinical spaces**	In all modes, will there be sufficient privacy for a clinical consultation to occur? Will there be appropriate lighting and bandwidth for example in kiosks or the pharmacy for mode 2 and 4.
**13. Clinic templates and coding for TMAT**	What adjustments need to be made to current clinical templates and coding? Do new codes need to be developed, for example to reflect mode of TMAT delivery?
**14. Further information**	Either the patient or the clinician may need further information. For example, it may not be possible on the first consultation for the clinician to be certain about withdrawal symptoms. Also, the patient may wish to have written information about MAT choices. In the case of sending information- this could be done by

DRAFT PLEASE DO NOT DISTRIBUTE

	way of email. With regards to withdrawals - the clinician may need additional information or ask for a healthcare worker visit to carry out drug testing, Dry Blood Spot Testing (DBST) for blood borne viruses such as HCV, venepuncture or a physical assessment. This is less of a problem in modes 3 & 4.
**15. Make contingency plans for what to do if something goes wrong (technically or clinically)**	Your service will need to have a contingency plan for possible technical problems or clinical problems. Technical problems may include data or privacy breaches, software or hardware breakdown as well as access and privacy difficulties. Clinical problems can be categorised as either mental health emergencies (for example an unexpected safe-guarding incident where a patient is going through a psychotic episode or threatening self-harm) or physical health emergencies. For example, though NHS Near Me is very stable, callers may run out of data, have a flat batter or get the wrong link. In this situation a good contingency plan is usually to revert to a phone consultation.
**16. Put arrangements in place for in-person contact where needed**	Examples of this may include prescriptions, medication, mobile phones/tablet devices, lab samples, food parcels, injecting equipment or naloxone

DRAFT PLEASE DO NOT DISTRIBUTE

Comments

[redacted] (process 1) equity of access

[redacted] (process 1) what about non healthcare workers e.g. role of peer support/volunteers/recovery workers?

[redacted] (process 1) What about wider engagement support e.g. IT

[redacted] (process 1) Worth adding any regulations to consider e.g. GDPR, CQC etc.

[redacted] (process 3) Please consider grouping related criteria together for example Clinical aspects (comorbidity, pregnancy, previous response etc.)/Social and Personal circumstances (remote location, poor transport access, Local Covid restrictions, access to technology, etc.)

[redacted] How will TMAT incorporate Family Inclusive Practice (FIP). During lockdown families report to us a significant decrease in the involvement in the treatment and care of their loved one due to use of technology to engage with them. FIP should be embedded in TMAT from the outset and encouraged

[redacted] (process 5) The names of the hub modes become very confusing for both patients and support professionals.

Specialist pharmacist in substance misuse: (process 5) Triadic Home – could a further option here be that the other worker is not actually at the home of the patient but joining the call between Prescriber and patient from another location or venue? This offers a different risk profile for alone working / visiting patients homes.

[redacted] (process 8) Clinician to patient calling should be on Near Me. This is agreed national platform. Other platforms don't have the necessary information governance or security stuff in place.

[redacted] (process 8) You can also consider drop in video clinics.

[redacted] (process 8) In Ayrshire addiction services, we have Near Me. We do a lot of review via telephone.

[redacted] (process 9) also thinking about non NHS services

[redacted] (process 9) Links should be sent via SMS or email (sending by letter or verbally will lead to miss-typing and the patient ending up in the wrong place). Alternatively, the link may also be embedded on an easily accessible webpage that the patient is directed to.

[redacted] (process 14) not just withdrawals though - may want to rephrase

[redacted] (process 14) drug testing? DBST? Venepuncture? Physical assessment?

[redacted] (process 15) Though Near Me is very stable – callers may run out of data/have a flat battery/get the link wrong so good contingency plan (usually reverting to phone consultation is required)

[redacted] (process 15) access and privacy considerations

[redacted] (process 15) better to rephrase as mental health or physical health emergencies which can includes lots of different scenarios.

[redacted] (process 15) TMAT needs to be contactable by more than just phone. We have seen a considerable decrease in people accessing upport using the phone. Initial requests for support need to be able to be made via email, online, text (non voice based contacts) which are free and ease to enrole

[redacted] (process 15) signpost to reputable sources e.g. C&M website

[redacted]

- Family inclusive practice (FIP) should be encouraged in every interaction with a person who uses substances. This needs to be built into the process to ensure that families are included in the treatment and care of their loved one.
- How are TMAT consultations going to take place to include family members. Families have told us during lockdown they have found it incredibly difficult to be part of their loved ones treatment and care due to the use of technology. They would normally attend appointments in person along with their loved one. During lockdown and the use of technology families have been excluded. This TMAT process does not promote FIP and actively excludes families.
- How will family support be offered to those involved in their loved ones TMAT treatment and care? Rights Respect & Recovery states that families have the right to support in their own right not merely as part of their loved ones treatment and care.

DRAFT PLEASE DO NOT DISTRIBUTE

2. Risk, safeguarding and ethical considerations

Risk-assessment process.

Understanding the risks associated with each step in the provision of Telemedicine Medication Assisted Treatment (TMAT) and conducting an appropriate risk assessment is good practice and is advised across the development of healthcare services. This process, called Clinical Risk Management (CRM), adopts a number of risk management tools from other industries to use in clinical situations enabling a logical process to understand why critical incidents occur and create reasonable mitigation strategies (Rausand, 2013; Kaya, Ward & Clarkson, 2019). Risk management tools include Failure Mode and Effects Analysis (FMEA) and Root Cause Analysis (Senders, 2004). Most if not all NHS services have established processes and so this guide does not elaborate on this further. Nevertheless, one useful exercise to guide further risk assessment activities is to conduct process mapping.

Process Mapping

DRAFT PLEASE DO NOT DISTRIBUTE

Process maps are a standard tool in business and engineering which attempts to visualise the workflow of a particular activity in order to improve efficiency (King, Ben-Tovim, Bassham, 2006). The flow of events in a process is mapped out such that we can delve into the mechanics of how intended and unintended outcomes happen. By identifying areas that have gone wrong or disrupted efficiency, we can introduce improvements. Process maps also enhances communications within a management structure by aligning understandings of the processes and standardises documentation (Mould, Bowers & Ghattas, 2010).
We have attempted in this section to breakdown each step in MAT to enable us to start thinking about how telemedicine may be used and what risk exposure may arise as a result. Figures 3 & 4 can be adapted to suit your own services and potentially shorten the process of logically breaking down the important steps of Medication Assisted Treatment (MAT) delivery.

Comments

[redacted]nterim Senior Charge Nurse: How do patients of no fixed abode / no access to phone / internet etc access treatment and appointments?

[redacted]ies[redacted]fected by alcohol & drugs:
Accessing the service (how accessible is the service?): Services need to be contactable by non voice based contacts (online, email, webchat, text). Through lockdown we have seen an increase in non voice based contacts for people initially enquiring/looking to access support.
Accessing the service (how inclusive and safe is the service?): Family Inclusive Practice needs to be included in this section. How will service users be pro actively encouraged to include family/friends in their treatment and care. Family involvement should be actively encouraged.
Identification: Reference made to next of kin but no mention of family/friends network.
Treatment planning: Again no mention of how families will be supported to be part of their loved ones treatment and care. How are we making sure that families are supported and have access to Naloxone? (Families are entitled to support in their own right. This is not the same as Family Inclusive Practice. Practitioners should be identifying appropriate supports for the families.
Titration: If family are included in the care and treatment of their loved one this will be an avenue of enquiry if people miss appointments or the practitioner has concerns. Families can be a real wealth of knowledge regarding how a loved one is coping outwith appointment times. With appropriate family support families can ensure that their loved one attend their appointments. Issue of Naloxone for families should be a priority.

Specialist pharmacist in substance misuse:
Stabilisation Co-morbidities: I think clearer recommendations re BBV testing, treatment (and the need for repeated testing later) should be made
Relapse: Can it be re-inforced the need for overdose awareness and risk plus supply of naloxone, at what can be a very risky time for the individual.

Figure 3: In-person MAT Service Process Mapping A (12–23)

Process	Activity			Risks
Access	How do service-users find you?<br>• Word of mouth/ peers<br>• Internet/ social media<br>• Other agency referral<br>• Advertisements<br>• Outreach<br>• Hospital in-reach<br>• Freephone number	How accessible is the service?<br>• Distance/ travel costs<br>• Opening times<br>• Appointment or drop in<br>• Out of hours access<br>• Referral only<br>• No wrong door	How inclusive & safe is the service?<br>• Psychologically informed<br>• Welcoming & friendly<br>• Trustworthy & private<br>• Transparent & respectful<br>• Peer support<br>• Collaborative<br>• Promoting choice & voice<br>• Gender specific options<br>• Language	• Easily ignored or marginalised groups may not access the relevant information<br>• Stigmatising or disrespectful attitudes may discourage people from attending<br>• Rigid opening times or appointment times may not suit this group<br>• People with no fixed address will not receive postal appointments<br>• Delays in MAT is associated with increased DRD, IVDU, BBV transmission<br>• 40-50% of people who Have died from DRD were not in treatment
Enrollment	Enrolment<br>• Identification<br>• Contact details<br>• Registered GP<br>• Receiving a prescription elsewhere?<br>• Eligibility<br>• Registering on your service system<br>• Next of kin<br>• Other supports including social work	Past Medical history<br>• Access to medical records<br>• Treatment (MAT) history<br>• Recent investigations including LFT, drug tests, ECG<br>• Non-fatal overdose history<br>• Co-morbidities<br>• Prescribed medication<br>• Allergies<br>• Medication Contraindications	Additional needs<br>• BBV testing<br>• IEP<br>• Naloxone<br>• Primary care<br>• Mental health support<br>• Financial support<br>• Housing<br>• Food & clothing<br>• Family welfare<br>• Legal support<br>• Advocacy<br>• Dispensing pharmacy	• Delays in the enrolment processes may delay safe prescribing, increasing the risk of withdrawals, DRD, IVDU & BBV transmission<br>• Opiate withdrawals are independently associated with receptive syringe sharing & non-fatal overdose<br>• Failure to conduct these checks may result in double prescribing or harms such as overdose from unsafe prescribing<br>• Missing opportunities to provide other supports such as harm reduction or benefits maximisation exposes the patient to ongoing risks at a critical time in their treatment
Diagnosis	Assessment<br>• Substances, preferred mode (sniff, smoke, inject), daily use (amount, cost), desired effect (up, down, feel normal)<br>• Tolerance, withdrawal<br>• Age at first use<br>• Strengths/skills<br>• Social support<br>• Criminal justice history<br>• Drug mixing, infection risk, NFO, psychiatric symptoms, prior treatment experience<br>• DSM V, ICD10 criteria	Examination<br>• Pulse, BP<br>• Tremor, agitation<br>• Temperature<br>• Pupil size<br>• Sweating, yawning, irritability<br>• Mental state<br>• Injection sites<br>• Jaundice	Investigations/ standardised tools<br>• Toxicology (near patient oral swab or urine testing)<br>• LFTs &/or ECG<br>• BBV near patient testing/ DBST or serum sampling)<br>• Statutory dataset collection for example NDTMS or SMR25A)<br>• Other tools such as COWS or SOWS	• A diagnosis of opioid use disorder is an absolute prerequisite to initiate MAT<br>• An overestimation of a patient's level of tolerance to opioids may result in an overdose<br>• Where the necessary information is collated , it is possible to make a diagnosis of dependence & make a prescribing decision at the first appointment.<br>• Patients already well-known to the service can be safely re-assessed and re-started on treatment rapidly<br>• Arbitrary attendance at multiple appointments before initiating treatment increases risks of harm to the patient.
Treatment planning	Review potential harms<br>• OD/ death<br>• Infections<br>• Social isolation<br>• Acquisitive crime<br>• Incarceration<br>• Drug debt<br>• Violence<br>• Exploitation<br>• Homelessness<br>• Family breakdown<br>• Suicidality	Explore patient goals<br>• There is an ethical & legal obligation upon care providers to ensure that the patient's values and priorities are understood and considered in clinical decision-making<br>• Use same I Reduce harms<br>• Use less I Reduce harms<br>• Stop using I Abstinence<br>• Moving on<br>• Rehabilitation (residential or otherwise)<br>• Stability<br>• Personal development	Support an informed treatment choice<br>• Mechanism of action of each MAT option (typically methadone or buprenorphine)<br>• Pros & cons of each MAT option<br>• Debunk myths<br>• Explain pre-requisites (for example COWS > 8-10 & gap from last opioid use for conventional buprenorphine induction<br>• Make recommendation based on risk-benefit ratio & patient preference	• Credibility, trust & rapport is required for treatment recommendations to be accepted<br>• A mismatch between clinician & patient outcome goals can result in disengagement in services<br>• Organisations are not held accountable in statutory & ethical obligation to support informed choice and to take account of patient values<br>• Patients may not be supported to claim their human rights to health

DRAFT PLEASE DO NOT DISTRIBUTE

Figure 4: In-person MAT Service Process Mapping B(12–23)

Process	Activity			Risks
Induction	**Safety**<br>• Identity confirmation<br>• State at time of induction (Intoxication, withdrawals, neutral) and the associated physical examination.<br>• Breath alcohol level if indicated<br>• What follow up arrangements should be made?	**Medication specific issues**<br>• Buprenorphine - lower risk of respiratory depression & overdose. Unobserved home induction possible. However, conventionally, requires definite withdrawals.<br>• Methadone - start low, go slow. Greater risk with unobserved dosing of overdose/diversion	**The first dose**<br>• Where will dispensing occur?<br>• Who will supervise the dose?<br>• Is the patient required to be observed again some time after first dose?<br>• What about depot formulations?	• Both the clinician and the dispensing pharmacy will need to confirm identity<br>• With poly-substance use and more then one dependency, patients may need to have take alcohol or benzodiazepines to attend safely. Guidance around breath alcohol levels or intoxication can help decision-making. Being in withdrawal may deter attendance.<br>• Alternative methods of buprenorphine induction are off license.<br>• Some depot formulations require dispensing to a clinician who will then administer.
Titration	**Attendance**<br>• Frequency of face to face encounters with a prescriber (daily, twice a week, weekly)<br>• Are reviews done by proxy?<br>• How will non-attendance be managed?<br>• How will loss to follow up be managed?<br>• What is done to increase retention in treatment?	**Support**<br>• Travel expenses, childcare<br>• Outreach<br>• Appointment reminders<br>• Tracing if appointments missed<br>• Peer or psychosocial support<br>• Ongoing harm reduction interventions such as naloxone	**Dosing related issues**<br>• Frequency of dose increases<br>• Contingency planning with missed doses<br>• Guidance on managing intoxication when attending pharmacy	• The risk of death is raised at the beginning and end of treatment<br>• Methadone initiation requires prescribing at sub-therapeutic doses and subsequent titration to a therapeutic dose over many weeks, resulting in risks with ongoing drug use and death. This makes the first 4 weeks of treatment risky.<br>• There is a period of very high mortality risk in the first few months immediately after treatment cessation, which is at least eight times higher than the mortality risk during treatment<br>• Supervised consumption is used to reduce the risk of OD and diversion and is associated with a reduction in illicit heroin and alcohol use but is associated with decreased retention in treatment<br>• Mortality risk at treatment onset is lower among those initiated on to buprenorphine than methadone, but retention may be better with methadone.
Stabilisation	**Co-morbidities**<br>• UK studies have reported co-occurring substance use (SUD) and mental health disorders at rates of 20–37% across all mental health settings and 6–15% in addiction settings<br>• There is an association between SUD and blood borne virus transmission, cardiovascular and respiratory disease, chronic pain, accidents & injuries<br>• Stabilisation may provide opportunities for these co-existing conditions to be diagnosed and managed<br>• Treatment of HIV, HCV & TB have clear public health benefits	**Managing others substances**<br>• Poly-drug use including illicit benzodiazepines such as etizolam, IV use or crack cocaine use and co-existing high risk alcohol consumption needs to be managed.<br>• High-risk opioid use is typically associated with benzodiazepine use to self-medicate, increase the effects of heroin or methadone. Illicit benzodiazepines are also taken to treat symptoms of psychiatric disorders, negative emotional states and withdrawal symptoms.	**Psychosocial interventions**<br>• Contingency Management, Cognitive Behaviour Therapy, Relapse Prevention, Dialectical Behaviour Therapy, Group Drug Counselling, mutual aid groups including 12-step & SMART, employment support, education & training, up-skilling, benefits maximisation, legal support, advocacy, compassion focussed therapy, mindfulness	• Opportunities to provide patients undergoing MAT support with other co-morbidities & co-occurring poly-substance use has great benefits to the patient and the general public. Equally, barriers to MAT & associated health protecting opportunities can endanger health for example by enabling viral transmission & TB spread.<br>• High threshold services placing obligations on patients to participate in psychosocial therapies before being allowed access to MAT have low levels of engagement & retention.<br>• MAT which does not introduce avenues to stabilisation of co-occurring poly-substance use may not result in improved outcomes<br>• Cessation of illicit substances may uncover psychiatric issues which need management & support
Medication reviews	**Adapting to changing circumstances**<br>• Is the service cognisant of changing health circumstances for example onset of cardiovascular or respiratory disease as the patient ages.<br>• Is the service sensitive and responsive to changing patient goals and the potential for a widening gap between this and service goals?<br>• Is the service competent in adjusting prescribing for example in situations of palliative care or acute pain<br>• Can prescribing arrangements be adjusted rapidly to meet family obligations or holiday plans	**Managing relpases**<br>• How will relapses be managed?<br>• Are responses punitive or supportive?<br>• Is the re-initiation of MAT in someone falling of treatment straightforward ?<br>• Are harm reduction interventions readily available in a non-judgemental way?<br>• Are outreach services in contact with people at risk of relapse?	**Moving forward**<br>• Is the service adjusting the duration and frequency of supervised consumption?<br>• Does the service provide or link in with other services that support moving forward in life if the patient wishes it?<br>• Are there opportunities to access MAT that reduces the need for pharmacy contact for example long acting formulations	• Long-term supervised dosing for stable patients is often inappropriate and a waste of resources. It runs the risk of impeding patients from normalising their lives, or dropping out of treatment prematurely.<br>• Services that do not adapt to changes in the life course or do seek the best holistic health outcomes for their patients run the risk of contravening their human rights to health and autonomy

DRAFT PLEASE DO NOT DISTRIBUTE

When working through your own Medication Assisted Treatment (MAT) process map, consider the following.

- Will offering Telemedicine Medication Assisted Treatment (TMAT) in one or more of the 4 modes increase or decrease risk when compared to conventional services and also when compared to contextual factors such as delays in service provision due to capacity issues or the pandemic?
- What steps can be taken to resolve the identified risk? In particular, would changing the mode or intake criteria alter this risk?
- Is the risk of using TMAT greater than not seeing the patient at all?
- Are TMAT risks any different to in person risks? What in reality is offered in-person which meaningfully reduces the risk in providing MAT?
- What other types of risks (or new forms of risk) might there be – such as data protection or privacy?
- If TMAT is used in only one particular group for example for patients undergoing 3-monthly medication reviews, are there processes to use the increased appointment capacity to shorten waiting times for in-person evaluation and induction?

The outcome of the process mapping and initial risk assessments should be clear and easy to follow safeguarding contingency plans including a 'what to do if…' plan in the event of an emergency or concern during a virtual appointment.

A wide range of low to high probability risk scenarios with a range of impact levels should be considered. Staff will be able to relate more closely to scenarios which are applicable to their setting, and as close to real-life circumstances as possible and so make better use of the contingencies and processes.

DRAFT PLEASE DO NOT DISTRIBUTE

Table 2 TMAT Risks and Actions

TMAT specific risk scenario	Actions to consider
Virtual settings should mirror in-person appointment settings.	With the pandemic, many healthcare workers have been working from home. It is important that the patient continues to feel that they are receiving a highly professional service which may involve the clinician ensuring that they have a home office which is appropriate. Also, most in person settings are private and the consultation should not be overhead. This will need to be mirrored in kiosk or virtual pod settings.
Information governance-consultation platform	Patients may already be very familiar with FaceTime and WhatsApp and wonder why the service is making things seem complicated. Unfortunately, many of these platforms are not compliant with healthcare standards. In Scotland, NHS Near Me is often used.
Information governance-data storage	Patients may have concerns over whether recordings will be made and distributed and what the risks are to breaches in confidentiality. The clinician will need to be able to confirm end-to end encryption robustness if asked for reassurance.
Informed consent to a virtual consultation	Informed consent is required from any person who is receiving a video consultation. Implied consent usually applies in in-person consultations- the person has attended in person indicating they wish a consultation with you. This is not quite as clear cut with virtual consultations. Explicit consent where the clinician states the activities involved in the consultation, ensures the person has the capacity to understand and has understood the explanation and agrees to proceed is recommended here.
Informed consent to Medication Assisted Treatment (MAT)	A further layer of difficulty may arise if the clinician cannot be certain that the patient has fully understood the implications of certain decisions or medication options- strategies to support these issues are detailed below.

Comments

When working through your own Medication Assisted Treatment (MAT) process map, consider the following.

Frances: does this need to be more generic? Rather than specify pandemic- hopefully, it won't always be a consideration! e.g. Other contextual factors, eg. the pandemic?

3. A guide to conducting the TMAT consultation

This final section describes some possible modes of TMAT delivery and how the video consultation may take place depending on the mode selected.

Modes of TMAT

Figure 5. Mode 1 and Mode 2 of TMAT delivery

1. Hub-home: Clinician connects from clinic to patient at home.

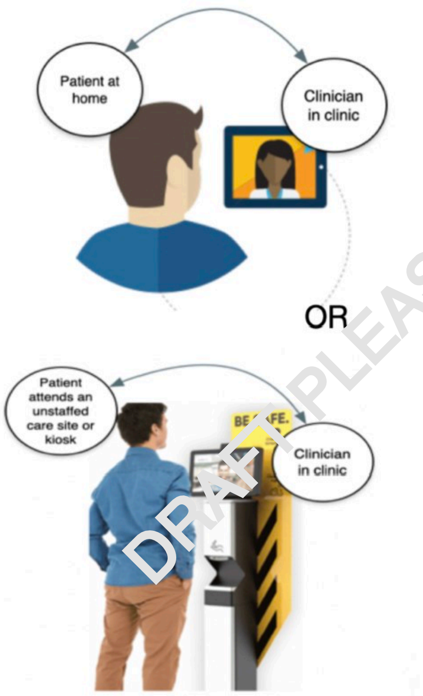

2. Dyadic hub-spoke: Clinician in specialist hub centre connects to patient in remote spoke healthcare site without additional staff member present (e.g. in an unstaffed kiosk).

Process	Considerations
Access	**Highly accessible.** Where the patient has internet access and a smartphone/tablet or laptop/pc, there is no need for travel (Mode 1). In the absence of this, the option of unstaffed pods or kiosks may require some travel to a nearby location. Another option may be for a mobile phone to be loaned to the patient for the appointment.
Enrolment	Visual confirmation of **identity** possible. This can be done by an administrator who could also carry out other checks. **Harm reduction advice** can be provided, and IEP, naloxone and near patient testing for BBV can be sent **by post** to the patient.
Diagnosis	The clinician would need to rely on the patient taking their **own clinical observations** for example with a saturation probe. Depending on lighting and camera quality, pupil size may be determined. ECG, blood tests or toxicology would usually require the patient to attend a health center
Treatment planning	Some strategies to improve comprehension such as illustrating an explanation may be impractical
Induction	How does the patient access the medication?
Titration	Dose increases can be discussed virtually, however, a prescription must still be generated & the patient needs to then access the medication
Stabilisation	Services such as benefits maximisation, virtual mutual aid, counselling or other forms of therapy can be provided virtually.
Medical review	3 monthly reviews may still require the patient to provide a drug test. Dose or dispensing arrangement changes still requires a prescription to be generated.

DRAFT PLEASE DO NOT DISTRIBUTE

Figure 6. Mode 3 and Mode 4 of TMAT delivery

3. Triadic hub-spoke: Clinician in specialist 'hub' centre connects to patient in remote 'spoke' healthcare site (for example, a community pharmacy, GP practice) with another healthcare worker present (for example, nurse, pharmacist, healthcare support worker)

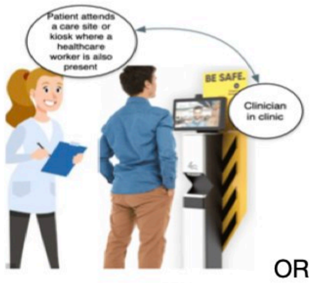

OR

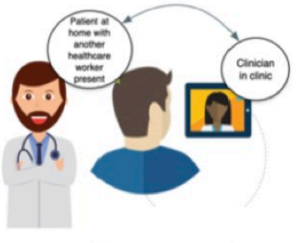

4. Triadic hub-home: Clinician in specialist 'hub' centre connects to patient at home with another healthcare worker present (for example, nurse, GP, healthcare support worker)

Process	Considerations
Access	**In Mode 4:** It is possible for people who are housebound needing additional support to use telemedicine or engage virtually with the clinician to be supported at home by a healthcare worker. This worker could also bring in the required technology to the patient's home. In **Mode 3,** a healthcare worker can help a patient once they get to their local access point such as a community pharmacy.
Enrolment	Identity checks, registration & preliminary work can take place through the healthcare worker
Diagnosis	**Clinical observations, standardised form such as COWS, toxicology testing** can all be done by the healthcare worker before the telemedicine consultation begins.
Treatment planning	The healthcare worker is able to sense check understanding
Induction	The healthcare worker will be able to collect and deliver medication. If the "spoke" site is in a pharmacy arrangements can be made in advance for medication to be dispensed and supervised at point of contact.
Titration	Similarly dose review appointments may occur virtually with healthcare worker supporting prescription related issues.
Stabilisation	Services such as benefits maximisation, virtual mutual aid, counselling or other forms of therapy can be provided virtually.
Medical review	3 monthly reviews may occur virtually with healthcare worker supporting prescription related issues.

DRAFT PLEASE DO NOT DISTRIBUTE

Comments

[redacted] affected by alcohol and drugs: Again no mention or reference to family inclusive practice. A guide on how Family Inclusive Practice can be incorporated should be standard. If it is not promoted from the outset workers will opt out for families to be involved. This has become highly evident during lockdown.

[redacted] amend healthcare worker to reflect SMS – see prev slide comment

[redacted] ok – nice clear slide BUT doesn't match the title – this isn't a guide to conducting consultations

The TMAT Consultation

Figure 7. TMAT consultation using Mode 1& 2: Diagram adapted from the Welsh National Video Consultation Service Toolkit (TEC Cymru. Video Consulting Toolkits [Internet]. Digital Health Wales, 2021).

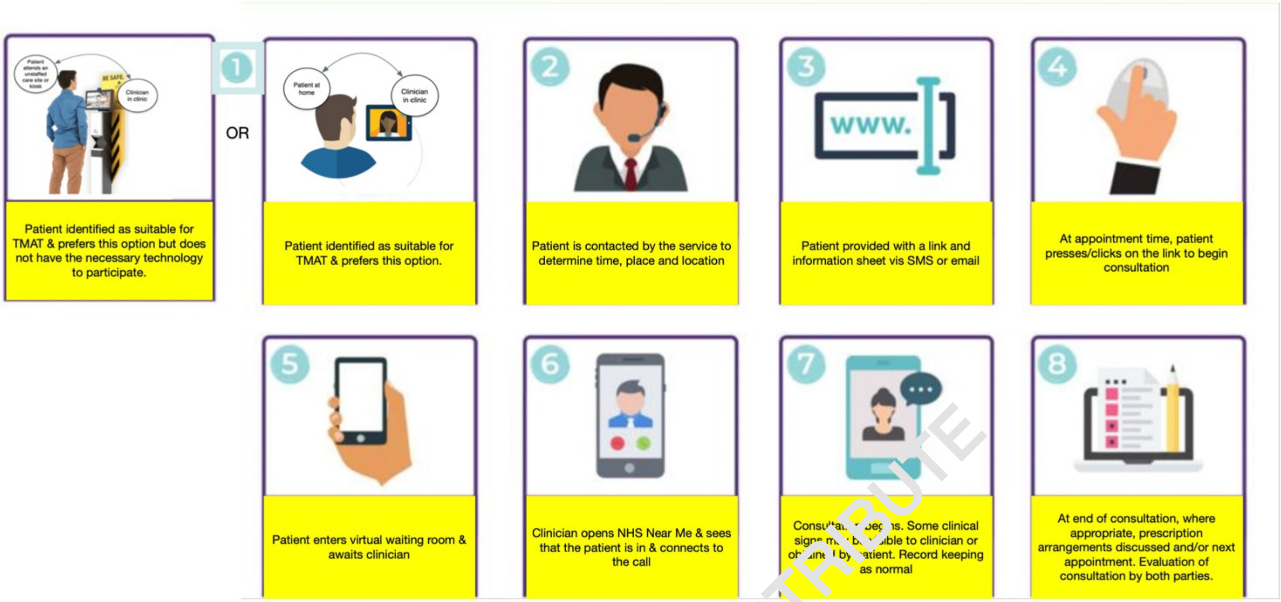

Figure 8. TMAT consultation using Mode 3 & 4: Diagram adapted from the Welsh National Video Consultation Service Toolkit (TEC Cymru, 2021).

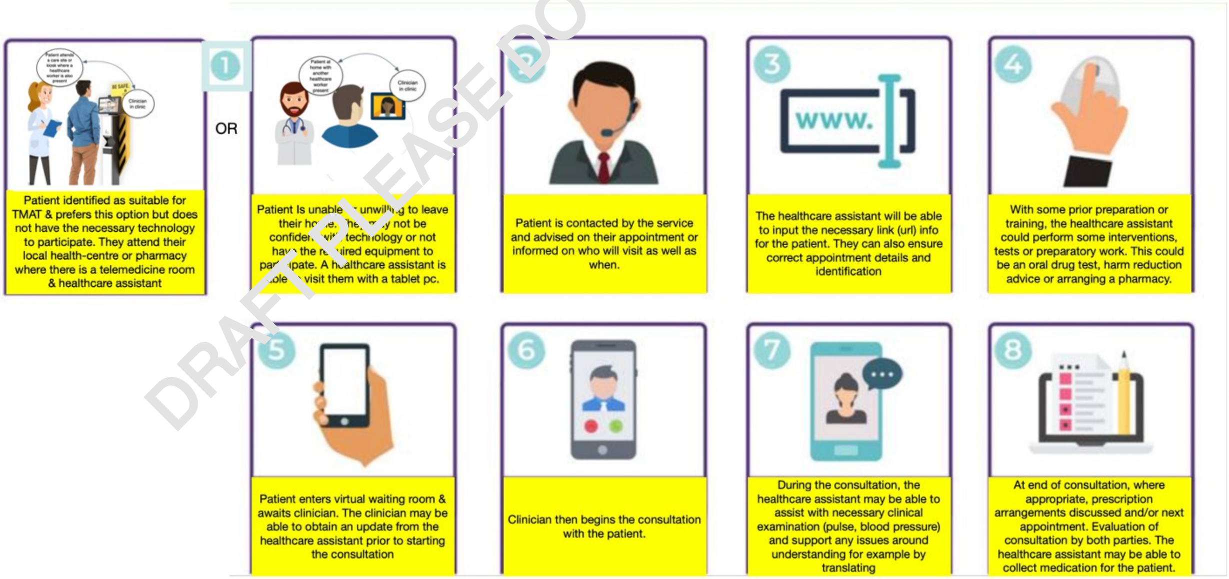

DRAFT PLEASE DO NOT DISTRIBUTE

Evaluation component

This project is specifically intended to empower and enable addiction services to develop Telemedicine Medication Assisted Treatment (TMAT) as a quality improvement endeavour. Implicit in this therefore is an iterative cycle of evaluation and improvement. There are several guides and frameworks to support this (Healthcare Improvement Scotland, 2018; Backhouse & Ogunlayi, 2020; Jones, Vaux & Olsson-Brown, 2019; NHS Institute for Innovation & Improvement, 2005) and many NHS boards have a dedicated quality improvement resource. A recent review of questionnaires evaluating telemedicine services (Hajesmaeel-Gohari & Bahaadinbeigy, 2021) identified that in order to optimise future telemedicine interventions, it is essential to focus on service-user needs, end-user acceptance, implementation processes and service-users' satisfaction. Table 3 lists some of the more common questionnaires available to evaluate aspects of telemedicine. The DigitAS team are currently developing an implementation evaluation questionnaire based on Normalisation Process Theory (Gillespie et al., 2018) for telehealth and telemedicine interventions.

TABLE 3 COMMON QUESTIONNAIRES USED IN TELEMEDICINE EVALUATION

Questionnaire name	Purpose	Reference
Telemedicine Satisfaction Questionnaire (TSQ)	Evaluating satisfaction with telemedicine	(Yip et al., 2003)
Telehealth Usability Questionnaire (TUQ)	Evaluating the usability of the telemedicine service	(Parmanto et al., 2016)
Service User Technology Acceptability Questionnaire (SUTAQ)	Evaluating the acceptance of telemedicine	(Yip et al., 2003)
The Telemedicine Service Maturity Model	Evaluating the implementation of telemedicine services	(Madhavan, 2013, p. 249)

DRAFT PLEASE DO NOT DISTRIBUTE

Comments

[redacted] any plans to collate any associated evaluation/QI work?

[redacted] Not just NHS! Need to ensure not NHS centric across all boards

[redacted] check wording throughout all boards - service users or patients?

[redacted] Much has already been done to evaluate Near Me – need not to re-invent the wheel. Need to look at published reports on Scot Gov website (evaluation reports, pre and post covid written by U of Oxford, public engagement report and EQIA).

Conclusions

This draft document outlines a simple visual step-by-step guide to help addiction services to set up TMAT. Addiction services are among the most regulated systems of care largely due to the regular prescribing of controlled drugs. It is therefore no surprise that there is a lag in the adoption of technological solutions to address capacity issues in treatment delivery compared with other health services. The perceived need for telemedicine mediated treatment has increased in the pandemic context, with a greater willingness to test new ways of working. In our exploration of the telemedicine landscape in addiction services, we found several examples of accelerated adoption of TMAT in North America, Australia and Ireland. Further, we have seen successful outcomes from the first ever feasibility study of Telemedicine in Addiction Services in the UK (Mayet, 2019). With rising drug related deaths in Scotland, in keeping with rates in North America, it is critical that we find ways to bridge this telemedicine lag. We conclude this guidance with a final overarching infographic in Figure 9 illustrating what a TMAT service may look like.

Comments

[redacted] Conclusions could perhaps be a bit more tightly worded, with a few key points?

Figure 9 comments:

Appropriateness and suitability box

[redacted] so that is basically all stages of the treatment pathway – so not sure about the need to mention this?

[redacted] hmmm not sure about `safer' – more about is it suitable for managing associated risks or not

[redacted] perhaps amend aware of e.g. indemnity issues. Maybe rephrase about e.g. interventions can appropriately be implemented via TMAT, the person is unable to use/access/
Adequate support not available to enable?

Red flags box

[redacted] rephrase: distress is relative so maybe that this mode would cause otherwise avoidable distress, unable to adequately complete required physical assessments

Workflow box

[redacted] (workflow) what about signposting to national guidance e.g. from GMC: **https://www.gmc-uk.org/ethical-guidance/ethical-hub/remote-consultations**

Box 6

[redacted] what about the person's choice and shared decision making – TMAT should be an OPTION in addition to others

[redacted] what about access to devices to enable appropriate examination e.g. automated blood pressure, other people like carers or recovery staff able to undertake?

DRAFT PLEASE DO NOT DISTRIBUTE

Figure 9: A visual guide to TMAT. Diagram adapted from the Welsh National Video Consultation Service Toolkit (TEC Cymru, 2021)

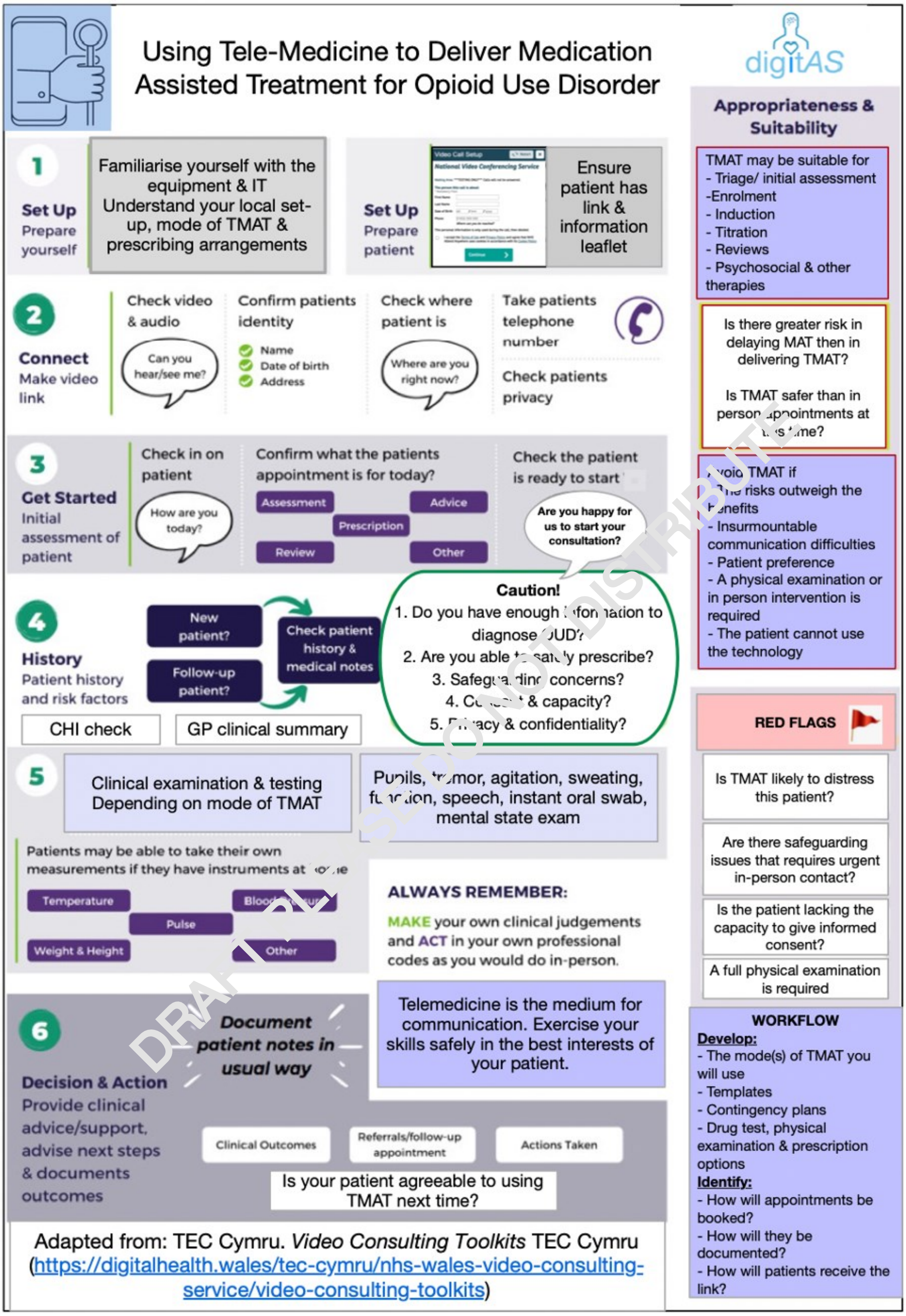

Supplement: Multimedia Appendix 4 [file mental_v10i1e47186_app4.pdf]
