# Supplementary material for: Developing a Theoretically Informed Implementation Model for Telemedicine-Delivered Medication for Opioid Use Disorder: Qualitative Study With Key Informants
Source: JMIR Ment Health. 2023 Oct 18;10:e47186. doi: 10.2196/47186 (PMC10620637; doi:10.2196/47186)

digitAS-TMAT

Best Practice Guidance for the Design & Delivery of a Telemedicine Medication Assisted Treatment (TMAT) Service for Opioid Use Disorder (OUD)

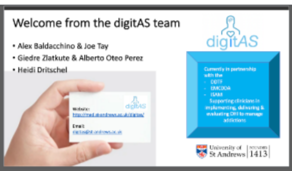

1
1 board

Introduction
1 board, 5 cards

2
1 board

Purpose of the guide
1 board, 3 cards

3
1 board

Methodology
2 boards, 5 cards

Step by step guide
1 card

Sections 4 to 7 describes TMAT delivery from three perspectives.

Section 4 describes a set up process and considerations around TMAT delivery in an existing healthcare workflow.

Section 5 discusses the ethical and legal principles to be considered in TMAT delivery.

Section 6 discusses the actual TMAT consultation.

Section 7 provided by our colleagues Roz Gittins & Claire Morrison takes us through some of the pharmacy considerations when delivering TMAT.

Section 8 briefly identifies a few examples of evaluation tools for your intervention.

We use tables and diagrams extensively to make this guide more easily adapted to local needs and to make information more readily available when required.

4
1 board

Setting up your TMAT service
1 board, 32 cards

5
1 board

Risk, safeguarding & ethical considerations
3 boards, 5 cards

6
1 board

A guide to conducting the TMAT consultation
3 boards, 5 cards

7
1 board

Pharmacy considerations
1 board, 13 cards

8
1 board

Evaluation
1 board, 3 cards

9
1 board

Conclusions
1 board, 3 cards

References
1 board

References
1 board, 17 cards

We have attached some of the slides from the case studies presented at the TMAT seminar here as well as the reference list for this guide.

Case studies
1 board

Case-studies
7 boards

Purpose of the guide

Purpose of the guide

Home

11 boards, 4 cards

Roz 14 May 2021
Explain TMAT abbreviation

Heidi 19 May 2021
TMAT - Telemedicine Medication Assisted Treatment

Figure 1. The Medication Assisted Treatment (MAT) standards mapped against Telemedicine Medication Assisted Treatment (TMAT) delivery.

frances 10 Jun 2021
Agree re: put acronyms in full and clarification re: numbers. Not sure what the final format will look like and who the target audience is?

We see [illegible] on of Telem[illegible]) in keeping with t[illegible]atment (MAT) standa[illegible]or the MAT standards themselves which have been reported elsewhere (Johns et al., 2020). Figure 1. maps out the relationship between the MAT standards and the various aspects of TMAT delivery. The knowledge and literature around the implementation of specific healthcare interventions are not always readily accessible in the public domain. Specifically, when it comes to technology based interventions, or interventions in response to crises, the published literature becomes rapidly outdated (Mohr et al., 2015). We have therefore taken the Digital Design for Addiction Services (Colley & Marttila, 2017, pg 295-8) approach which is described in more detail below.

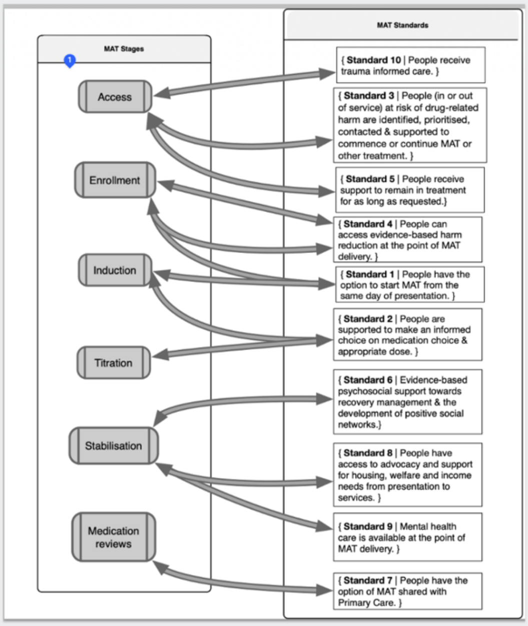

Supplement: Multimedia Appendix 5 [file mental_v10i1e47186_app5.pdf]
