# Supplementary material for: Developing a Theoretically Informed Implementation Model for Telemedicine-Delivered Medication for Opioid Use Disorder: Qualitative Study With Key Informants
Source: JMIR Ment Health. 2023 Oct 18;10:e47186. doi: 10.2196/47186 (PMC10620637; doi:10.2196/47186)
Supplement: Multimedia Appendix 6 [file mental_v10i1e47186_app6.docx]

### Initial stakeholder engagement and ongoing participation.

| Stakeholder role | Engagement event: active chatroom participation. | Document commenting and revisions | Final roundtable discussion |
| --- | --- | --- | --- |
| Drug Deaths Task Force (Scotland) | 1 | 1 | 1 |
| Director of pharmacy | 1 | 1 | 1 |
| Addictions Research Fellow | 1 |  |  |
| Peer/ Person with lived experience | 1 | 1 |  |
| Families of PWUD (advocacy group) |  | 2 |  |
| Service innovation lead | 1 | 1 | 1 |
| Scottish Government  (Digital Innovation) | 1 |  | 1 |
| Technology Enabled Care Scotland |  | 1 | 1 |
| Third sector organisation(s) | 1 | 1 |  |
| Addiction Service Nurse Managers | 4 | 1 | 1 |
| Addiction Psychiatrists | 4 | 1 | 2 |
| General Practitioners | 3 | 1 | 1 |
| Post-doctoral researchers | 2 | 3 | 3 |
| Addictions service clinical director | 6 | 1 | 2 |
| Senior Addiction Nurse | 1 | 2 | 1 |
| Pharmacists | 2 | 1 | 0 |
| Total | 28 | 18 | 15 |
