# Supplementary material for: Developing a Theoretically Informed Implementation Model for Telemedicine-Delivered Medication for Opioid Use Disorder: Qualitative Study With Key Informants
Source: JMIR Ment Health. 2023 Oct 18;10:e47186. doi: 10.2196/47186 (PMC10620637; doi:10.2196/47186)
Supplement: Multimedia Appendix 7 [file mental_v10i1e47186_app7.docx]

Multimedia Appendix 7. Examples of inclusion and exclusion criteria for offering TMOUD

**Individuals appropriate for TMOUD (without an in-person component)**

- Identity has been verified, or person already known to the service
- Follow up or clinical review
- Triage appointment
- The patient is accompanied by an outreach worker, care navigator or facilitator who has been trained to work in this TMOUD model
- The patient is receiving care in a shared-care environment (for example in an emergency department or primary care facility)

**Individuals who may be appropriate for hybrid TMOUD (with an in-person component).**

- New to treatment
- A drug test is required
- Individuals who have recently relapsed
- A history of medication non-adherence
- Missing multiple telehealth visits
- Experiencing homelessness
- A co-morbid condition requiring a physical examination or laboratory testing

**Relative contraindications to TMOUD**

- Unable to verify identity
- Situations where in-person contact is highly desirable for example complex medication needs, poly-substance use or concerns around medications diversion
- Unwilling or unable to attend recommended telehealth appointments (e.g., no access to devices to support telemedicine)
- Patient wishes to have an in-person appointment
