# Supplementary material for: Developing a Theoretically Informed Implementation Model for Telemedicine-Delivered Medication for Opioid Use Disorder: Qualitative Study With Key Informants
Source: JMIR Ment Health. 2023 Oct 18;10:e47186. doi: 10.2196/47186 (PMC10620637; doi:10.2196/47186)
Supplement: Multimedia Appendix 8 [file mental_v10i1e47186_app8.docx]

###### Multimedia Appendix 8. Clinic co-ordination and administration requirements

1. **The appointments system and privacy**

- Identify how TMOUD appointments will be booked and documented.
- Identify the person(s) in charge of making the TMOUD appointments. They should counter check that contact information such as email addresses or mobile numbers for short message service (SMS) are correct to avoid the link for the appointment going to the wrong person.
- How much in advance can TMOUD appointments be booked? This is particularly relevant in populations which may not have consistent mobile phone numbers or addresses.
- Provide a range of ways for people to access the appointment link including via an SMS, text or email, or the link may be embedded on a webpage that person is directed to.
- Telemedicine booths may be an appropriate way to provide privacy to people who are experiencing homelessness or have no privacy at home.
- How will appointment slots be offered, documented, and given to the delivering clinician? Does the system used to deliver TMOUD integrate well with existing appointment systems?

1. **Setting up the clinical space**

- In all four models of TMOUD delivery, it is important to ensure that there will be sufficient privacy for a clinical consultation to occur.
- Pre-approved locations for example telemedicine booths or healthcare locations such as pharmacies need to have adequate privacy, lighting and bandwidth.
- Clinic templates and coding within existing medical records need to be adjusted for TMOUD consults. This may include the development of new codes to reflect the mode of TMOUD delivery, whether the full consultation occurred via telemedicine and how additional clinical investigations such as urine drug tests were obtained.

1. **Make contingency plans for what to do if something goes wrong (technically or clinically)**

- Contingency plans for possible technical problems which may include data or privacy breaches, software glitches, hardware breakdown, as well as access and privacy difficulties. While TMOUD platforms may be relatively stable, individuals may run out of data, be unable to charge devices or access the correct link. In this situation, a good contingency plan is usually to revert to a phone consultation.
- Contingency for clinical problems may include mental health emergencies (for example an unexpected safeguarding incident where a person is experiencing a psychotic episode or threatening self-harm) or physical health emergencies.

1. **Put arrangements in place for in-person contact where needed. This may be necessary to**

- Collect prescriptions
- Access mobile phones/tablet devices
- Drop off samples for analysis
- Collect food parcels, injecting equipment, or take-home naloxone

1. **Introduce the option of family inclusive practice**

- This should be an option in every interaction with a person who uses substances.
- For this to be a meaningful offer, services need to know in advance how TMOUD consultations can take place to include family members.
- The introduction of technology may exclude families from being part of a person’s treatment where previously they may have attended in-person consults.
