# Supplementary material for: Developing a Theoretically Informed Implementation Model for Telemedicine-Delivered Medication for Opioid Use Disorder: Qualitative Study With Key Informants
Source: JMIR Ment Health. 2023 Oct 18;10:e47186. doi: 10.2196/47186 (PMC10620637; doi:10.2196/47186)

Workflow process mapping to characterize the in-person delivery of medication for opioid use disorder

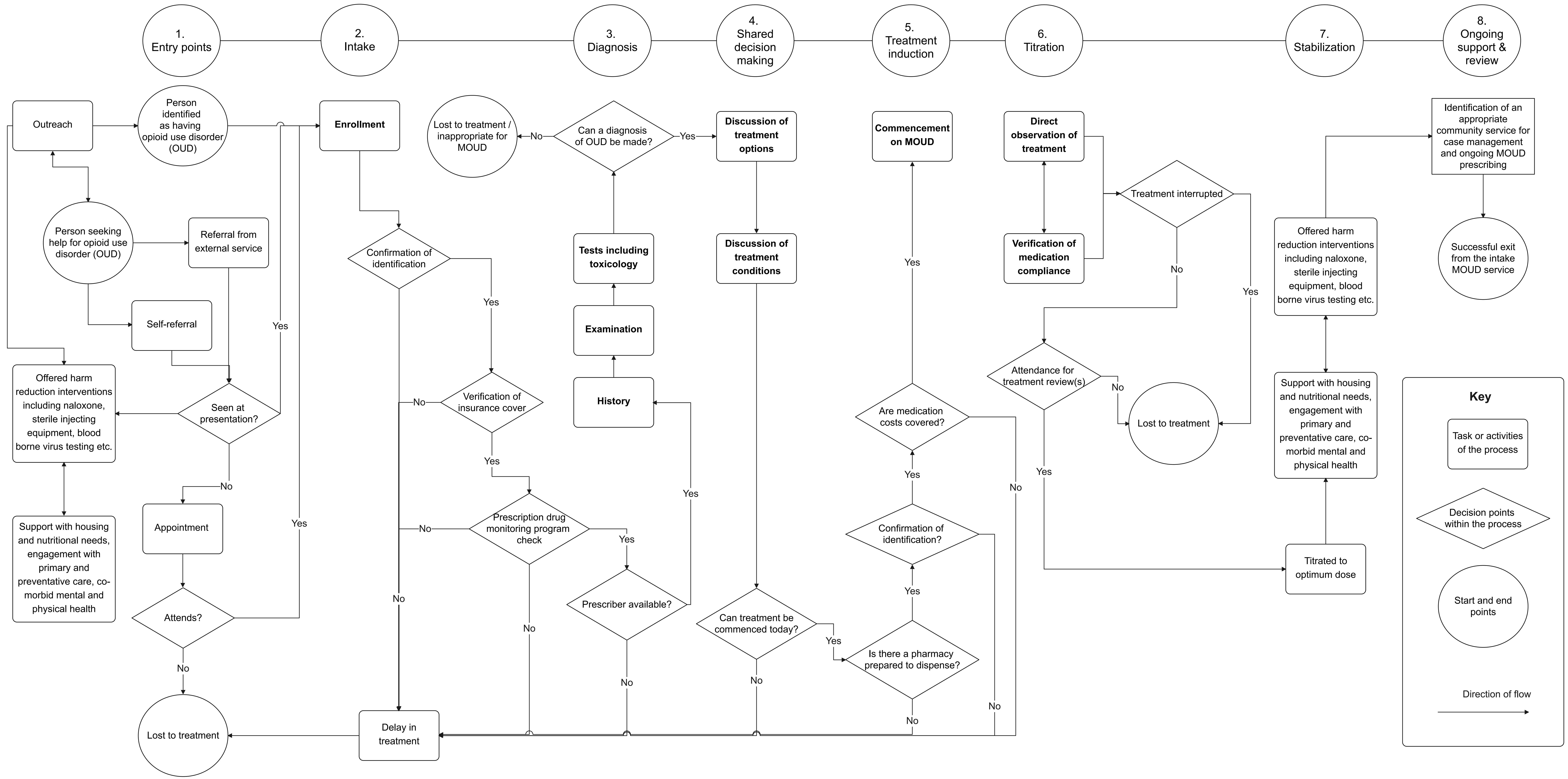

Supplement: Multimedia Appendix 10 [file mental_v10i1e47186_app10.pdf]
